# Effects of Grape and Grape-Derived Bioactives on Inflammation and Immune-Driven Mechanisms Within the Context of Cardiometabolic Health and Aging: A Review

**DOI:** 10.3390/nu18182952

**Published:** 2026-09-09

**Authors:** Indraja Gaddam, Marcela Santos Ferreira, Catherine Hastings, Mariana C. Calle, Catherine J. Andersen

**Affiliations:** 1 Department of Nutritional Sciences, University of Connecticut, Storrs, CT 06269, USA; 2 Department of Health Sciences, Worcester State University, Worcester, MA 01602, USA

**Keywords:** grapes, grape polyphenols, cardiometabolic disease, aging, inflammation, immune-mediated mechanisms, antioxidant, gut microbiome

## Abstract

Cardiometabolic disease and age-related health complications are significant clinical and public health concerns that impact an individual’s overall quality of life and longevity. Chronic low-grade and immune-mediated inflammation underlies the pathophysiology of metabolic and systemic tissue dysfunction associated with cardiometabolic disease development and accelerated aging, whereas therapeutic and preventative strategies to reduce inflammation and promote immune function yield beneficial health outcomes. Evidence from cell and animal studies demonstrates that consumption of grapes, grape-based extracts and preparations, and grape-derived bioactive compounds exert beneficial anti-inflammatory and immunomodulatory effects, which are associated with improvements in metabolic profiles. However, results from human intervention trials investigating grape effects on inflammatory and immune markers are more limited and report varied effects on improvements in clinical and experimental biomarkers indicative of cardiometabolic disease risk and healthy aging, such as serum lipids, blood pressure, insulin resistance, oxidative stress, gut dysbiosis, and cognitive function. Variability in responses to grape products in human trials may be attributable to differences in grape product preparation in addition to variability in the health status of the study population. In this review, we present an overview of mechanistic studies evaluating the effects of grapes and grape-derived products on inflammatory and immune pathways within the context of cardiometabolic disease and aging, and provide an update on recent intervention trials in this area.

## 1. Introduction

Chronic inflammation and immune dysregulation play key roles in the etiology and progression of several cardiometabolic diseases, including obesity, type 2 diabetes mellitus (T2DM), hypertension, cardiovascular disease (CVD), metabolic dysfunction-associated steatotic liver disease (MASLD), and obesity-associated asthma and other comorbidities, in addition to physiological changes underlying health complications associated with aging [1,2,3,4]. There has been a steady increase in the prevalence of chronic conditions over the past 20 years that is expected to continue, which represents major and growing public health concerns worldwide [5]. Mechanistically, these conditions are characterized by low-grade chronic inflammation, increased production of pro-inflammatory cytokines and oxidative stress, hyperinsulinemia, insulin resistance, dyslipidemia, endothelial dysfunction, and gut dysbiosis—all of which may contribute to disease progression and metabolic complications [6]. Earlier onset of metabolic dysfunction is associated with greater risk of age-related physical decline and comorbidities [7], which is a critical clinical and public health concern given that the population in the U.S. is aging, with 1 in 6 Americans classified as being 65 years or older according to the 2023 Census [8]. Thus, preventative and therapeutic strategies that mitigate inflammation and immune dysfunction may promote favorable clinical and public health outcomes pertaining to cardiometabolic disease and aging.

Lifestyle interventions, including adherence to healthful, nutrient-dense dietary patterns, have long been considered important for disease prevention and longevity [9,10]. Evidence from mechanistic and clinical studies demonstrates that bioactive-rich foods and their functional components can modulate disease-mediating immune and inflammatory pathways [11]. Grapes and grape-derived compounds confer a wide range of health benefits, with a growing body of research reporting effects that may directly and indirectly modulate inflammatory and immune mechanisms that play key roles in the prevention and management of cardiometabolic disease and physiological dysfunction associated with accelerated aging [12,13].

Grapes are one of the oldest and most widely cultivated fruits in the world. Botanically, they are classified as berries and can be eaten in different forms, e.g., fresh (table grapes), dried (raisins), fermented (wine), and juices/extracts (supplements). The main difference between table grapes and wine grapes lies in their size, skin thickness, and sugar content [14]. Table grapes are typically large, seedless, and thin-skinned, while wine grapes are smaller, thick-skinned, and contain a much higher sugar concentration, making them well suited to fermentation [15]. Raisins are conventionally produced from varieties such as Thompson seedless, which have high sugar content and no seeds [16]. The global production of grapes has shown continuous growth over the past twenty years. In 2000, grape production was estimated at 63.6 million tonnes (MT) from 7.25 million hectares of cultivated land, and this had increased to 75.9 MT by 2024, despite a decrease in harvested area to 6.7 million hectares [17]. The principal producers of grapes worldwide are China, Italy, France, and the United States [18]. Along with the increase in production, the consumption of grapes and their products has also changed over the last two decades, with the consumption of table grapes increasing more than fivefold from 2000 to 2024 (14.8 MT to 31.7 MT), consistent with the growing worldwide demand for fresh fruit. In contrast, the consumption of dried grapes decreased slightly (from 1.34 MT to 1.22 MT), while the consumption of wine remained stable [19].

Nutritionally, grapes are low in calories, primarily consisting of water and carbohydrates. According to the US Department of Agriculture (USDA) FoodData Central database, the composition of green and red or pink table grapes differs slightly, with 100 g of red or green grapes being approximately 74.5 Kcal energy, 0.9 g protein, 0.2 g fat, 19.4 g carbohydrates, of which 16.7 g are total sugars, and nearly 90% water by weight [20,21]. Grapes are relatively rich in vitamin K and copper, while also providing modest amounts of potassium, phosphorus, calcium and vitamin C [20,21]. In dried grape products such as raisins, the nutritional profile differs, with vitamin B2 being the most abundant, followed by vitamin B6, while vitamin C is present in lower amounts [22]. Vitamin E can be extracted from grape seed oil derived from wine residues [23]. Grapes are also a rich source of bioactive phytochemicals, especially polyphenols, that have antioxidant, anti-inflammatory, cardioprotective, and immunomodulatory properties [24]. The distribution of these polyphenols differs considerably across different parts of the grape. The total phenolic content (TPC), a measure of overall concentration of phenolic compounds, is highest in the seeds, followed by the skin, with the pulp containing the least [25]. The polyphenols also differ based on grape variety, color, growing conditions and post-harvest processing [26]. Polyphenols found in grapes are divided into two classes: flavonoids (consisting of flavonols, flavan-3-ols, flavones, flavanones and anthocyanins) and non-flavonoids (such as stilbenes and phenolic acids (hydroxybenzoic acids and hydroxycinnamic acids)). Among the flavonoids, flavan-3-ols are the most abundant in the skin and seeds of grapes [27] and barely detected in the pulp [24]. The main monomeric flavan-3-ols are catechin and epicatechin, among others, such as gallocatechin and epigallocatechin. These form galloyl derivatives with gallic acid, and they can also be polymerized to form proanthocyanidins, occurring chiefly within the seeds and skin [28]. Flavonols represent the second most abundant group of flavonoids in grapes and are accumulated mainly in the skins, and in some varieties, they have been found in seeds. The major flavonols are quercetin, kaempferol, myricetin, and isorhamnetin. Derivatives of quercetin, like quercetin-3-O-glucoside and quercetin-3-O-glucuronide, are predominant in both red and white varieties, whereas derivatives of myricetin are found in the red varieties only [29,30]. Anthocyanins are water-soluble pigments found chiefly in the grape skin and are responsible for the characteristic pigmentation, such as red, purple, and blue. Their contents and composition vary according to grape varieties, vineyard practices, and environmental factors like the season and the place of growth. The predominant anthocyanins to be found in grapes are the derivatives of delphinidin, cyanidin, peonidin, petunidin, pelargonidin, and malvidin, the latter being the most abundant [31]. In addition to these, certain Teinturier varieties like Rubired have the anthocyanins deposited in both skin and flesh, a feature not found amongst the common varieties [28]. It is important to note that red grapes can produce anthocyanins, while white varieties cannot [12].

Among the non-flavonoid polyphenols, stilbenes and phenolic acids have significant biological activity. Stilbenes are a class of phytoalexins that serve as a defense mechanism of the plant, and the main stilbenes found in grapes are resveratrol occurring as both the cis and trans isomeric forms, in addition to the glycosylated derivative form of piceid (resveratrol-3-O-β-D-glucopyranoside), piceatannol, and resveratrol oligomers (viniferins). These compounds are mostly found within grape skins, roots, and leaves [32]. The phenolic acids may be divided into hydroxybenzoic acids and hydroxycinnamic acids [33]. The predominant hydroxybenzoic acids are gallic acid, syringic acid, protocatechuic acid, gentisic acid and ellagic acid, and among hydroxycinnamic acids, caffeic acid, chlorogenic acid, *p*-coumaric acid, ferulic acid, and sinapic acid are most notable. These are all chiefly found in the skin, although some can also be found in pulp or seeds, with their concentration influenced by grape varieties and growing regions [33].

**Figure 1:**
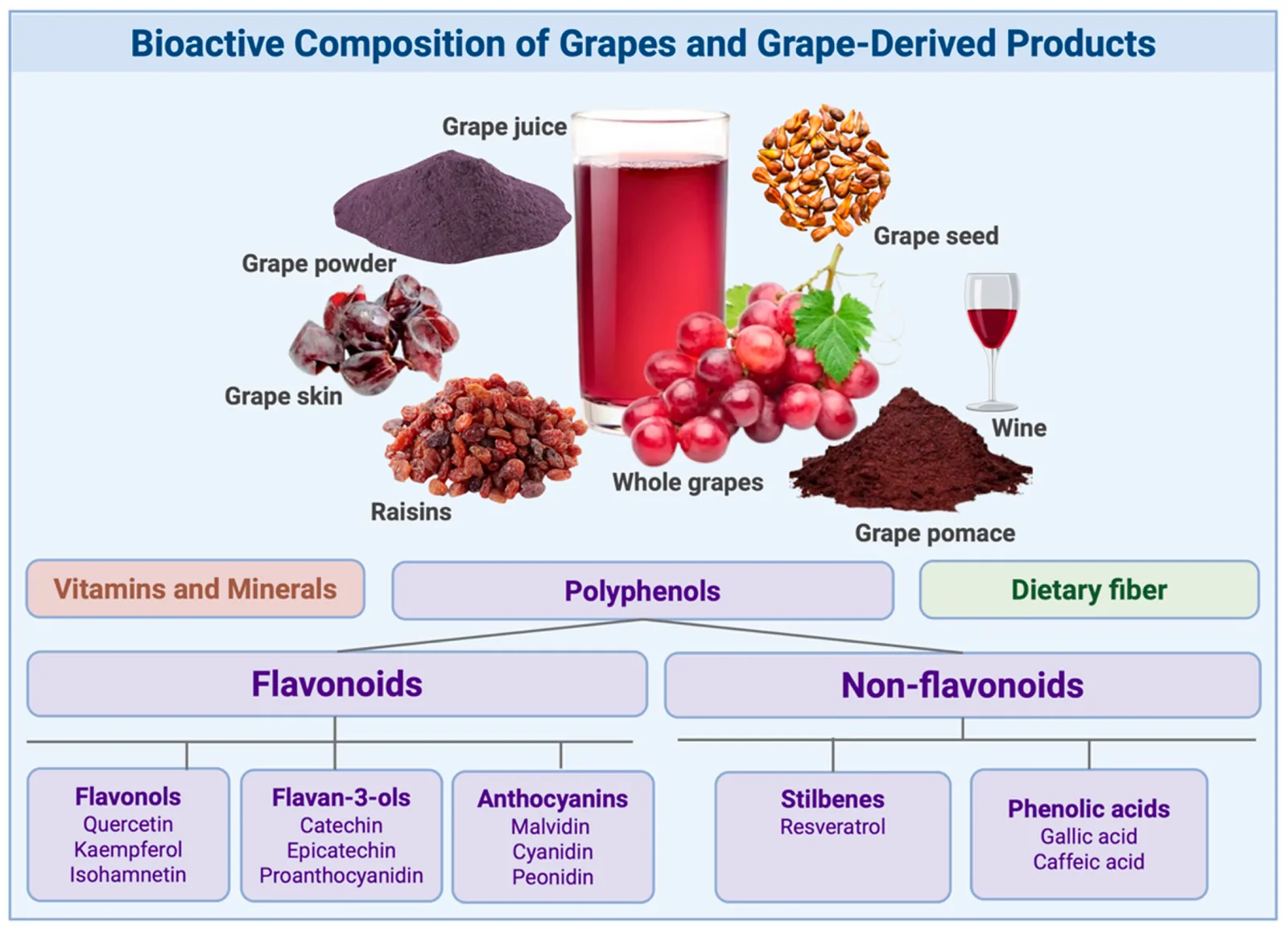
Bioactive composition of grapes and grape-derived products. Grapes and grape-derived products contain a variety of bioactive constituents, including polyphenols, dietary fiber, and vitamins and minerals. Representative polyphenol subclasses and compounds commonly found in grapes are shown. Created in BioRender. Andersen, C. (2026) https://BioRender.com/r51q6wt.

Beyond whole grapes, grape-derived products such as grape juice, wine, grape pomace, freeze-dried grape powder, and grape seed extracts have been widely used by both mechanistic and intervention studies due to their high polyphenol content. About 75% of fresh grape production is used for making wine through fermenting juice, out of which approximately 20–30% is the byproduct called grape pomace, consisting of skin, seeds, stalks, and remaining pulp [34]. Interestingly, about 70% of polyphenols are retained in the pomace after wine processing [34]. Since grape seeds contain the highest number of polyphenols, especially proanthocyanidins, grape seed extracts and grape seed oil have also become increasingly popular in many studies, owing to their antioxidant, anti-inflammatory, and immunomodulatory properties [35]. A summary of the nutrients and bioactive polyphenols found in commonly studied grape products is shown in Figure 1.

Evidence from cell culture, animal, and clinical studies suggests that grapes and grape-derived bioactives exert beneficial effects on cardiometabolic health and aging through multiple interconnected mechanisms. Experimental studies have shown that various grape products and grape polyphenols may modulate chronic inflammation, oxidative stress, immune dysfunction, insulin resistance, inflammaging and gut microbiota composition [36,37,38,39,40,41]. Accordingly, in in vitro and animal studies, grapes and grape-derived compounds mediate improvements in systemic inflammation, endothelial dysfunction, oxidative stress, immune regulation, and gut barrier integrity in different models of obesity, cardiovascular disease, metabolic dysfunction and aging [42,43,44,45,46]. Human clinical trials have also shown beneficial effects on oxidative stress, metabolic dysfunction, lipid metabolism, glucose regulation, and cognitive function; however, findings remain variable depending on grape products, dosage, intervention duration, and the population studied [47,48,49,50]. Overall, current evidence shows the potential cardiometabolic and immunomodulatory benefits of grape-derived bioactives, although recent clinical trials (e.g., within the last 5 years) addressing immune outcomes are lacking, and well-controlled clinical studies are needed to better understand the translational relevance to human health and healthy aging.

This review will highlight studies evaluating the effects of grapes and grape-derived bioactives on inflammation and immune function within the context of cardiometabolic health, chronic disease, and aging, with a focus on the mechanistic action of grape compounds and evidence from recent clinical intervention trials. To conduct this review, PubMed and Scopus were used as primary databases within which to identify primary research publications based on search terms such as grape(s), grape powder, grape polyphenols, grape-derived bioactive, grape seed extract, grape seed oil, grape pomace, grape juice, raisins, currants, wine, anthocyanin, and resveratrol combined with inflammation, inflammaging, immune function, T cells, macrophages, cellular senescence, gut microbiome, intestinal barrier, oxidative stress, antioxidant, health effects, healthy, obesity, CVD, metabolic syndrome, insulin resistance, diabetes/T2DM, MASLD, and older/aging. Literature searches were conducted from June 2025 to April 2026. While a more comprehensive review of the literature is discussed for mechanistic studies, this review limits discussion of clinical trials to the last 5 years (July 2020–present), given the availability of other reviews that address studies on this topic in preceding years.

## 2. Role of Inflammation and Immune-Driven Mechanisms in Cardiometabolic Disease

Cardiometabolic diseases—such as obesity, T2DM, hypertension, CVD, and MASLD—are among the major causes of morbidity and mortality around the world [51,52]. Persistent chronic low-grade inflammation, a hallmark of cardiometabolic diseases, plays an important role in the initiation and progression of disease development [52]. Within the context of cardiometabolic disease, chronic, low-grade inflammation often originates from and exacerbates adipose tissue dysfunction-associated weight gain and obesity, leading to alterations in tissue and systemic immune profiles, dysregulation of lipid metabolism, and ectopic deposition of lipids that promotes progression of atherosclerosis and metabolic and immune tissue dysfunction; impairment of glucose metabolism and insulin resistance; and endothelial dysfunction, leading to impaired vasodilation and vascular remodeling [53]. The following section highlights key pathways in the pathophysiology of cardiometabolic diseases, with an emphasis on pathways known to be modulated by grapes and grape-derived bioactives.

### 2.1. Adipose Tissue Dysfunction and Inflammation in Cardiometabolic Disease Pathophysiology

Among the many factors that contribute to disruption of tissue homeostasis under inflammatory conditions, adipose tissue—particularly visceral adipose tissue (VAT)—plays an important role. Besides storing fat, VAT cells secrete important molecules that regulate homeostasis in different tissues [54]. VAT is composed of adipocytes, preadipocytes, fibroblasts, vascular cells, and both innate and adaptive immune cells. These cells act in synergy to secrete pro- and anti-inflammatory molecules, including adipokines, cytokines, and chemokines, to maintain homeostasis by balancing pro- and anti-inflammatory signals [54,55]. The function and differentiation of cells in VAT are influenced by hormones, such as adipokines, and intracellular sensors, including AMP-activated protein kinase (AMPK). AMPK acts as a metabolic regulator, monitoring energy status and detecting changes in ATP status, thereby regulating lipid, glucose, and protein metabolism [56]. When activated under energy stress, AMPK phosphorylates important enzymes to inhibit anabolic processes, such as fatty acid and cholesterol synthesis, and promote catabolic metabolism, such as fatty acid oxidation and glucose uptake [57]. In obesity, AMPK activity is often reduced, leading to uncontrolled lipid synthesis, accumulation of triglycerides and insulin resistance [58]. Moreover, adipocytes undergo hypertrophy and hyperplasia, creating a hypoxic state, triggering cellular stress. These changes promote adipocyte apoptosis and initiate an inflammatory response [58]. As a result, the cellular composition of VAT shifts, impacting the function and differentiation of adaptive immune cells, especially T cells [56]. Evidence show that the regulatory T cell (Treg) population, a specialized subset of cluster of differentiation (CD) 4^+^ T cells that acts to maintain immune balance and prevent excessive inflammation, is reduced [59] while effector CD8+ T cells [60] and CD4^+^ T interferon-gamma (IFN-γ)-producing cells, such as T helper (Th) 1, are enriched in obese tissue [61]. Concurrently, under homeostasis, anti-inflammatory signals support polarization of macrophages toward an M2 anti-inflammatory phenotype and suppress M1 pro-inflammatory phenotype proliferation [54,55]. However, IFN-γ, produced by different immune cells, including Th1, skews the polarization towards the M1 phenotype, supporting recruitment of leukocytes from the bloodstream [56]. Further, macrophages cluster together forming crown-like structures, which engulf dead adipocytes and secrete other pro-inflammatory cytokines such as tumor necrosis factor (TNF)-α and interleukin (IL)-6, as well as chemokines like monocyte chemoattractant protein 1 (MCP-1). This results in a chronic inflammatory environment dominated by M1-polarized macrophages and pro-inflammatory cytokines [54,62].

At the molecular level, the inflammatory response is tightly regulated by an intracellular signaling pathway that determines the balance between pro- and anti-inflammatory responses systematically. Nuclear factor kappa-light-chain-enhancer of activated B cells (NF-κB) is a master regulator transcription factor related to the inflammatory response. Different stimuli activate NF-κB, including TNF-α, IL-6, and IL-1β [63]. Proteomic analyses have shown that chronic TNF-α exposure in adipocytes upregulates key proteins in the NF-κB pathway involved in immune cell recruitment, potentially increasing the inflammatory response [64]. TNF-α additionally stimulates lipolysis in adipocytes, breaking down triglycerides and releasing free fatty acids (FFAs) in the bloodstream, which further activate macrophages via Toll-like receptor (TLR) 4 engagement, activating the NF-κB pathway, and maintaining the inflammatory status [56]. FFAs can be taken up by other tissues and when exacerbated contribute to lipid buildup in the liver, muscle, and pancreas [65]. Ectopic fat deposition promotes toxicity and impairs function and, together with pro-inflammatory cytokines, worsens tissue dysfunction, further contributing to the development of metabolic disorders [66]. In contrast, peroxisome proliferator-activated receptors (PPARs), a subfamily of ligand-activated nuclear receptors, counteract NF-κB, playing a regulatory role in lipid metabolism, glucose homeostasis, and inflammation, thus facilitating M2 polarization [67]. PPARα strongly suppresses other inflammatory pathways driven by activated protein-1 (AP-1) [57]. AP-1 comprehends a family of transcription factors that sense and respond to different stimuli, including cellular stress and reactive oxygen species (ROS) produced by inflammation. By binding specific DNA sequences, AP-1 regulates genes involved in cell growth, repair, and death. Chronic or excessive activation by ROS contributes to inflammation, aging, and the development of cardiometabolic diseases [68]. This balance between pro- and anti-inflammatory pathways is essential in determining the inflammation status in adipose tissue and other tissues [67].

In addition to promoting tissue dysfunction, chronic inflammation and oxidative stress may also modulate circulating lipoproteins. Dyslipidemia—characterized by abnormal circulating levels of total cholesterol, low-density lipoprotein cholesterol (LDL-C), high-density lipoprotein cholesterol (HDL-C), and triglycerides [69]—is a key metabolic consequence of chronic inflammation and adipose tissue dysfunction and is closely associated with increased cardiometabolic risk [70]. Moreover, under inflammatory conditions, high-density lipoprotein (HDL) particles may undergo compositional remodeling, characterized by the replacement of apolipoprotein AI (Apo-AI) protein by serum amyloid A (SAA), impairing their cholesterol efflux capacity and antioxidant properties. At the same time, ROS production and other inflammatory mediators, such as myeloperoxidase (MPO), promote the oxidation of low-density lipoprotein (LDL) particles, generating oxidized LDL (oxLDL), which is considered a highly atherogenic lipoprotein and closely associated with endothelial dysfunction, foam cell formation, and progression of atherosclerosis [71].

Collectively, these metabolic and inflammatory disturbances contribute to vascular dysfunction and hypertension. In a pro-inflammatory and pro-oxidant environment, circulating neutrophils, monocytes, and lymphocytes adopt a pro-inflammatory phenotype and may adhere to vascular endothelium, promoting leukocyte infiltration, further amplifying vascular inflammation and ROS production. An important source of ROS is the nicotinamide adenine dinucleotide phosphate (NADPH) oxidase family, which generates superoxide and hydrogen peroxide, contributing to oxidative stress, vascular remodeling, and impaired endothelial function. Importantly, oxidative stress is not restricted to the vascular endothelium but also affects the kidneys, where early ROS accumulation contributes to chronic injury, impaired sodium handling, and progressive blood pressure elevation [72].

### 2.2. Gut Microbiome Effects on Inflammatory Pathways and Immune Profiles Underlying Cardiometabolic Disease Pathophysiology

Beyond the adipose tissue, research over the past decade has revealed that gut microbiota has a tight influence on the immune system and metabolism. Gut and adipose tissue are interconnected through metabolic and immune pathways. Gut dysbiosis, characterized by reduced diversity of gut microbiota and growth of beneficial bacteria coupled with increased growth of unhealthy bacteria, is associated with the initiation and progression of different inflammatory diseases [73]. Studies in mice and humans show that obesity may drive dysbiosis by increasing gut permeability and supporting translocation of pro-inflammatory bacterial products, such as lipopolysaccharides (LPS), into lymph nodes and the bloodstream. High-fat diet (HFD)-fed mice, obesity models, and individuals with increased cardiometabolic disease risk often present with gut dysbiosis and a modified pro-inflammatory physiological environment, supported by increased macrophage and IFN-γ-producing cell proliferation, decreased Treg populations, and elevated concentrations of systemic inflammatory markers [74]. Importantly, higher concentrations of TNF-α and IL-6 have been found to be associated with a suppression of beneficial bacteria growth, whereas lower levels of these cytokines are correlated with a greater abundance of beneficial bacteria, supporting the tight correlation between inflammation and gut microbiome balance [73]. Additionally, gut microbiota have differential capacities to produce metabolites from the diet that can have both pro- and anti-inflammatory effects. For example, short-chain fatty acids (SCFAs), such as propionate and butyrate (which are predominantly synthesized from dietary fibers and complex carbohydrates) and KetoA, derived from linoleic acid, are preferentially produced by beneficial gut bacteria and have been shown to downregulate inflammatory cytokine production [75], promote shifts toward anti-inflammatory immune profiles through epigenetic and transcription regulation of pro-inflammatory cell surface receptors and the NLRP3 inflammasome, and associate with improved cardiometabolic risk profiles [76,77]. Moreover, bile acids play an important role in microbiota diversity and abundance, further impacting metabolic activity [78], whereas the gut microbiome also influences bile acid metabolism by modulating metabolites composition and activity, thereby regulating pathways related to inflammation, cardiometabolic diseases, and lipid homeostasis [79]. Importantly, gut bacteria profiles associated with obesity and cardiometabolic disease have a reduced capacity to generate cardioprotective and anti-inflammatory metabolites, including those derived from dietary polyphenols that are thought to mediate health benefits [80]. Although the underlying mechanisms are not fully understood, polyphenols can act as a bioactive compound in colon, modulating bacterial communication and virulence; alter membrane composition; and increase susceptibility to antibiotics. Importantly, it has been shown that they shift the microbiota towards beneficial taxa [81]. Further, polyphenols, such as resveratrol, have been shown to improve production of SCFA, whereas grape-derived complex carbohydrates and other components may serve as substrates for SCFA production, suggesting a bidirectional relationship between grape components and gut microbiota-derived metabolites [82]. Thus, the gut microbiota not only has an important role in contributing to the progression or prevention of cardiometabolic diseases but may explain differential, interindividual responses to dietary intervention.

### 2.3. The Role of Inflammatory and Immune-Mediated Pathways in Mediating Insulin Resistance

Together, alterations in gut microbiota composition and adipose tissue function propagate systemic inflammation and metabolic stress, which extend to insulin-sensitive organs. Due to ongoing inflammation and lack of resolution signals in the body, insulin resistance arises as a central metabolic disease linking adipose, hepatic, and intestinal dysfunction. Insulin resistance is defined as a condition in which tissue that usually responds to insulin becomes less sensitive, even when insulin levels are normal or elevated. Therefore, the glucose levels in the bloodstream are elevated. Persistent hyperglycemia leads to the development of T2DM [83]. Under normal conditions, pancreatic β-cells secrete insulin when glucose levels in the bloodstream are elevated. Insulin, in turn, travels to target tissues—such as muscle, liver, and adipose tissue—and binds its receptor, promoting glucose uptake [84]. Mechanistically, insulin binds to its receptor and activates the tyrosine kinase domain. Then, phosphorylation of insulin receptor substrate-1 (IRS1) occurs, acting as a binding site for phosphoinositide 3-kinase (PI3K), which in turn initiates a signaling cascade leading to activation of protein kinase B (AKT). This process results in glucose transporter 4 (GLUT4) translocation to the membrane surface, increasing glucose uptake, enhancing glycogen synthesis, and suppressing lipolysis and gluconeogenesis [85]. However, some cytokines can impact this process, resulting in decreased glucose uptake. TNF-α engagement to its receptor, tumor necrosis factor receptor 1 (TNFR1), triggers inflammatory signaling cascades that counteract insulin action [86]. Intracellular kinases, produced by the downstream signal of TNF-α, phosphorylate IRS1 at serine residue 307, inhibiting the normal function of IRS1. As a result, impairment of the PI3K/AKT signaling pathway occurs, leading to insulin resistance [85]. IL-6 further contributes to glucose homeostasis impairment by tagging IRS1 for ubiquitination. In a classic pathway, IL-6 binds to its receptor, IL-6R, expressed in the plasma membrane of hepatocytes and immune cells, leading to activation of Janus kinase (JAK), which in turn activates several signaling pathways, including phosphorylation of signal transducer and activator of transcription 1 and 3 (STAT1/STAT3) [87]. Phosphorylated STAT1/STAT3 moves into the nucleus and promotes transcription of target genes, including suppressor of cytokine signaling 3 (SOCS3), which marks IRS1 for degradation, consequently reducing insulin signaling [85]. Cellularly, prolonged insulin resistance and chronic inflammation place additional stress on β-cells, impairing their function over time [84]. Resident macrophages in pancreatic islets of Langerhans, specifically intra-islet macrophages, increase in number under the conditions of obesity. These macrophages engulf β-cell insulin secretory vesicles and lower insulin secretion. In parallel, IL-1β secreted by immune cells binds to the IL-1 receptor (IL-1R) highly expressed on β-cells, leading to NF-κB activation. As a consequence, a reduction of glucose-stimulated insulin secretion (GSIS) occurs [74]. Importantly, IL-1β is produced in response to NOD-like receptor family pyrin domain-containing 3 (NLRP3) inflammasome activation. Different stimuli can trigger the NLRP3 inflammasome, such as activation of TLR4 and elevated glucose levels, which further leads to oxidative stress and activation of the NLRP3 inflammasome. When activated, the NLRP3 inflammasome leads to activation of caspase-1, which in turn cleaves the inactive precursor molecule pro-IL-1β into its active form, thereby secreting IL-1β [88]. Altogether, these mechanisms contribute to the development of insulin resistance, declining glucose uptake, and persistent hyperglycemia that can lead to T2DM and increased risk of CVD and other cardiometabolic comorbidities.

### 2.4. Clinical Profiles Indicative of Inflammation and Immune Profiles Associated with Cardiometabolic Disease

As noted above, metabolic and tissue dysfunction give rise to altered clinical inflammatory and immune profiles that are routinely assessed for determining cardiometabolic disease risk in clinical and research settings. Systemic inflammatory markers, including high-sensitivity C-reactive protein (hsCRP) and circulating cytokines such as IL-6 [89] and TNF-α, reflect persistent low-grade inflammation in cardiometabolic disease [90]. In addition, systemic immune dysregulation is also reflected in the bloodstream, where elevated white blood cell counts have been identified as a simple and cost-effective marker of low-grade inflammation and have been associated with increased cardiometabolic risk [91]. Altogether, systemic inflammation and elevated pro-inflammatory cytokine levels promote Th17 cell differentiation, which is characterized by the production of IL-17, contributing to the inflammatory response. Furthermore, the disruption of Th17/Treg balance promotes a sustained pro-inflammatory state [92]. Importantly, this pro-inflammatory and immune-activated state is linked to increased oxidative stress, as discussed, and is also captured in clinical settings by circulating markers of lipid peroxidation, such as malondialdehyde (MDA), a stable biomarker reflecting systemic oxidative damage and lipid oxidation that has been associated with cardiometabolic outcomes [93]. It is important to note that many of these markers and cardiometabolic disease risk increase with aging, which is associated with similar pro-inflammatory and immune changes [94], which are reviewed in the following section.

## 3. Role of Inflammation and Immune-Mediated Mechanisms in Aging

Aging is characterized by chronic, low-grade inflammation, known as “inflammaging”, a natural, complex, multifactorial, and progressive phenomenon that occurs systematically over time [95]. The gradual accumulation of damage induced by different stressors—including environmental toxins, oxidative stress, chronic inflammation, and DNA mutations—leads to cellular and tissue dysfunction, ultimately triggering inflammaging [96]. This persistent inflammatory state is recognized as a key contributor to the onset and progression of numerous age-related diseases, such as neurodegenerative disorders, cancer, CVD, metabolic dysfunctions, and immune-related conditions [97]. Key mechanisms underlying this process include impairments in DNA repair, dysregulation of programmed cell death, alterations in immune activity, and sustained oxidative stress [98]. In addition, elevated circulating levels of IL-6, IL-1β, and TNF-α are positively correlated with cardiometabolic risk factors in older adults [99]. Moreover, elevated levels of pro-inflammatory cytokines and inflammatory markers, such as hsCRP, have been correlated to a decline in cognitive performance and can predict memory impairment, potentially due to oxidative stress and neuroinflammation [100]. In parallel, accumulation of oxidative stress during lifespan has been associated with the development of low-grade inflammation and age-related diseases, reinforcing the molecular and physiological hallmarks of aging [68]. The following section outlines the physiological inflammatory and immune-mediated changes that occur with aging, which may serve as important therapeutic targets to promote longevity.

### 3.1. The Role of Inflammation in Age-Related Tissue Senescence

In the context of inflammaging, several hallmarks emerge as key factors in mediating age-related decline in physiological and tissue function, including genomic instability, telomere attrition, epigenetic alterations, loss of proteostasis, deregulated nutrient sensing, mitochondrial dysfunction, stem cell exhaustion, altered intracellular communication, and cellular senescence [101]. Specifically, cellular senescence is characterized by a process in which cells undergo permanent growth arrest and lose their ability to respond to apoptotic signals [102]. Accumulation of senescent cells is known to impair local tissue function, as well as the development of systemic age-related diseases, including dysfunction in skeletal muscle, adipose tissue, heart, kidney, and stem cells [103]. Cell cycle arrest is mediated by two main pathways: inhibition via p16 or activation pathways through p53 and p21. These proteins are key regulators in cellular proliferation. Importantly, cellular senescence plays a dual role in physiology and aging. Under normal conditions, senescence is a protective mechanism, contributing to tissue repair and regeneration to recover tissue after injury [104]. However, senescent cells stay metabolically active and do not respond properly to apoptotic signals under inflammation and aging due to changes in gene expression, chromatin structure, increased lysosomal β-galactosidase activity (β-gal), and the development of the senescence-associated secretory phenotype (SASP) [105]. The SASP encompasses a range of bioactive molecules that influence the tissue microenvironment. These include pro-inflammatory cytokines and chemokines (IL-1β, IL-6, IL-8, IL-18, TNF-α, and MCP-1); matrix-degrading enzymes, such as matrix metalloproteinases; growth factors including vascular endothelial remodeling factors; various protein and non-protein substances, such as proteases, clotting factors, ceramides, and collagen; damage-associated molecular patterns (DAMPs); extracellular vesicles; microRNAs; DNA fragments; ROS; prostaglandin derivatives; and protein aggregates. Collectively, these bioactives act as signaling molecules that contribute to tissue dysfunction and activation of the NLRP3 inflammasome and NF-κB pathways [106]. Moreover, neutrophil and monocyte count rise with age, and phagocytic and chemotactic activity are impaired, potentially due to SASP factors release. Monocytes show higher levels of immune activation markers, lower expression of co-inhibitory molecules, and increased MCP-1 expression, contributing to inflammatory status [107]. Therefore, cellular senescence impairs the phagocytic and migratory abilities of macrophages and neutrophils, which under normal conditions engulf senescent cells and eliminate them [108].

### 3.2. The Role of Immunosenescence in Aging

Over time, this chronic inflammatory environment contributes to the progressive impairment of immune responses, impacting function and structure—a phenomenon referred to as immunosenescence [108]. Persistent inflammation and oxidative stress disrupt hematopoietic homeostasis. Hematopoietic stem cells (HSCs) differentiate into red blood cells, platelets, and leukocytes, maintaining immune and hematological homeostasis. However, HSCs undergo loss of self-renewal, and differentiation capacity is observed with aging, particularly reducing leukocyte output and decreasing immune responsiveness [109]. In addition, the thymus, a primary lymphoid organ essential for the formation of T cells and maintaining adaptive immune response, undergoes progressive atrophy during adulthood and involution in older individuals, representing an important hallmark of immunosenescence [110]. Some recent research in mice shows the thymus cell population seems to modify during aging, and a unique population was identified that emerges during aging, associated with decreased regenerative capacity [111]. Moreover, proper thymic function depends on crosstalk between thymus stromal cells and thymocyte development, both of which are indirectly dependent on NF-κB signaling for their formation and expansion. Furthermore, accumulation of adipocytes in the thymus over time impacts the function of the organ [112]. Together, these changes contribute to immunosenescence through lower output of T cells. Furthermore, the adaptive immune response affected by aging demonstrates weakened defense responses to infections and vaccines, reduced immunosurveillance that may facilitate tumor development, accumulation of memory T cells, and increased markers of senescence in T cells. Senescent T cells show decreased expression of different markers, including CD28, a co-stimulatory molecule crucial for the activation of T cells, impacting their proliferation capacity. As a result, T cells become dysfunctional, leading to weakened response to chronic infection, vaccines, and immunosurveillance [108].

### 3.3. The Role of Inflammation and Senescence in Driving Age-Related Changes in Metabolic Profiles

The consequences of immunosenescence extend beyond impaired immune responses, influencing metabolic regulation and tissue homeostasis. These immune alterations are closely linked to age-related changes in adipose tissue, which differ in characteristics and redistribution [102]. In terms of cell composition, aging has been associated with an increase in the number of M1 macrophages and a decrease in the M2 phenotype, similar to what is observed in obesity [113]. Simultaneously, body fat mass increases with aging, which contributes to the development of cardiometabolic diseases. One theory behind increased age-related adiposity suggests that stem cells under chronic stress differentiate into adipocytes instead of their normal lineage, contributing to ectopic fat deposition in the thymus, liver, muscle, heart, and blood vessels. This irregular buildup of fat triggers immune cell infiltration in those tissues, sustaining chronic low-grade inflammation [105].

Apart from adipose tissue dysfunction and its contribution to metabolic disturbances, other organs undergo metabolic stress. Pancreatic senescent β-cells secrete SASPs, particularly inflammatory cytokines and chemokines and extracellular matrix remodeling substances, leading to dysfunction and inflammation, resulting in a direct impact on insulin secretion and production in mouse models [114]. Concomitantly, skeletal muscle exhibits decreased glucose uptake, while the liver presents impaired gluconeogenesis and lipid accumulation. Together, these combined organ dysfunctions result in insulin resistance and hyperglycemia [115]. In addition, the gut environment undergoes changes in various aspects related to aging, such as pH, mucus layer, and immune signaling, thereby affecting the gut microbiome to support growth of harmful bacteria that further contribute to increased gut permeability and the release of LPS into the bloodstream, triggering systemic inflammation [116].

The cumulative effects of metabolic stress, systemic inflammation, and cellular senescence impact the vascular system, thereby accelerating vascular aging and atherosclerosis progression. Chronic accumulation of senescent endothelial cells and SASP production, especially cytokines and cell adhesion molecules (such as intracellular adhesion molecule-1 (ICAM-1) and vascular cell adhesion molecule-1 (VCAM-1)), in the cardiovascular system results in failure of cell apoptosis. These senescent cells persist in tissues, facilitating monocyte adhesion and migration into the vascular intima, contributing to the phagocytosis of oxidized LDL particles and the formation of foam cells. Vascular smooth muscle cells (VSMCs), which are important for the structural integrity of the vascular system, also undergo senescence and may change their phenotype to osteoblast-like cells. These cells release osteogenic factors, which are molecules that activate bone-related pathways within the vessel. This process leads to vascular calcification, the major risk for plaque instability, and is susceptible to rupture and thrombotic events [106].

Overall, aging and chronic inflammation demonstrate a complex interplay among metabolic organs, vascular tissues, and the gut microbiome, sustaining a pro-inflammatory environment that drives cardiometabolic disease risk and progression. Thus, preventative and therapeutic lifestyle and nutrition strategies that mitigate age-related inflammation, immune dysfunction, and senescence may reduce the burden of comorbidities and health complications in order to promote health and longevity [116].

## 4. Bioactive Properties and Mechanistic Effects of Grapes and Grape Components in Modulating Inflammation and Immune-Mediated Mechanisms in Cell and Animal Models of Cardiometabolic Health and Aging

It is well established that diets rich in nutrient- and bioactive-dense foods and their functional components can modulate inflammatory and immune pathways to reduce risk of cardiometabolic disease and age-related health decline [116,117,118]. Grapes, grape-derived products, and grape-derived bioactive compounds such as whole grape powder, grape seed oil, grape pomace extracts, and polyphenols such as resveratrol, quercetin, malvidin, catechins, and proanthocyanidins have been shown to modulate inflammatory pathways [33]. Grape-derived bioactives additionally exert modulatory effects on various immune cells such as monocytes, macrophages, and T cell subsets in pathological conditions, including HFD-induced obesity, atherosclerosis, hyperglycemia, and aging, where chronic low-grade inflammation disrupts innate and adaptive immune balance and inflammatory homeostasis [119,120]. Together, grape-mediated effects on inflammation and immune profiles have been associated with improvements in endothelial function, oxidative stress, and gut microbiota profile, thereby counteracting many of the mechanisms underlying cardiometabolic and age-related diseases [119,121,122].

**Figure 2:**
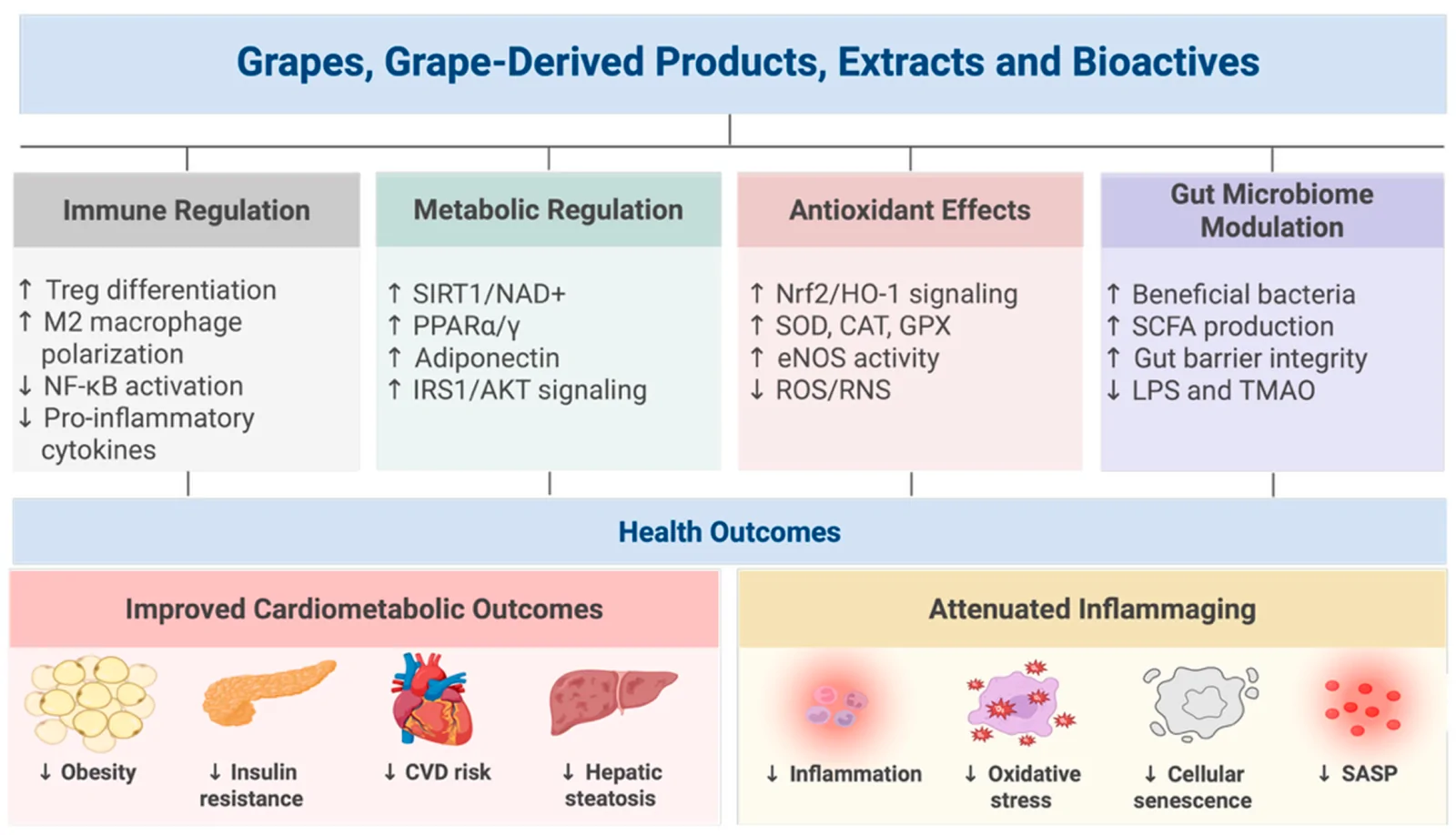
Overview of mechanistic effects of grape-derived bioactives in cell and animal models of cardiometabolic disease and aging. Grapes, grape-derived products, extracts, and bioactive compounds regulate multiple interconnected pathways such as immune cell modulation, metabolic reprogramming, antioxidant defense, and gut microbiome modulation. These effects are associated with enhanced Treg differentiation and M2 macrophage polarization, decreased systemic and tissue-specific inflammation, improved metabolic signaling, increased antioxidant capacity, and favorable alterations in gut microbiome. Together, these mechanisms contribute to attenuation of key cardiometabolic and aging disease outcomes such as reduced obesity, insulin resistance, cardiovascular risk, and hepatic steatosis, while attenuating inflammaging through reductions in inflammation, oxidative stress, cellular senescence and SASP. ↑: increase, ↓: decrease. Abbreviations: AKT: protein kinase B; CAT: catalase; CVD: cardiovascular disease; eNOS: endothelial nitric oxide synthase, GPX: glutathione peroxidase; HO-1: heme oxygenase 1; IRS1: insulin receptor substrate 1; LPS: lipopolysaccharide; NAD+: nicotinamide adenine dinucleotide; NF-κB: nuclear factor-κB; Nrf2: nuclear factor erythroid 2-related factor 2; PPARα/γ: peroxisome proliferator-activated receptor alpha/gamma; ROS/RNS: reactive oxygen/nitrogen species; SASP: senescence-associated secretory phenotype; SCFA: short-chain fatty acids; SIRT1: sirtuin 1; TMAO: trimethylamine *N*-oxide; Treg: regulatory T cell. Created in BioRender. Andersen, C. (2026) https://BioRender.com/zbn5w9s.

The following section outlines the effects of grapes, grape-based extracts and preparations, and grape-derived bioactives compounds on systemic and metabolic tissue inflammation and immune profiles in models of cardiometabolic disease (e.g., obesity, insulin resistance, hypertension, CVD) and aging in cell- and animal-based experimental models. This section further highlights mechanisms by which grapes and grape-derived components modulate inflammatory and immune pathways in cardiometabolic disease and aging models, with a focus on grape-mediated effects on oxidative stress and gut microbiome dynamics. An overview of grape effects on these mechanisms and health outcomes from preclinical models is depicted in Figure 2.

### 4.1. Effects of Grape-Derived Bioactives on Inflammatory T Cell Profiles in Experimental Models of Obesity

Obesity is associated with chronic low-grade inflammation, metabolic and lymphoid tissue dysfunction, elevated concentrations of circulating inflammatory markers, and pro-inflammatory T cell phenotypes, which are implicated in the pathophysiology of cardiometabolic disease and inflammaging [56,123]. Grape-derived bioactives, particularly resveratrol, have been shown to modulate T cell-mediated immune responses in diet-induced obese models. In C57BL/6 mice fed a HFD to induce obesity, a reduction in Tregs, characterized by CD4, CD25, forkhead box protein-3 (FOXP3), and increased expression of inflammatory markers was observed, potentially representing a shift towards more pro-inflammatory Th1 responses [39]. In contrast, resveratrol-treated obese mice showed an increase in the number of anti-inflammatory Tregs and IL-10 secretion and a decrease in pro-inflammatory IL-1, IL-6, and TNF-α in plasma and spleen [39]. Further, resveratrol-treated obese mice also showed a decreased ratio of TNF-α to IL-10, which may be indicative of a shift from a pro-inflammatory Th1 response to a Th2 anti-inflammatory response [39]. Splenic mRNA expression of Treg-related genes, such as *Foxp3*, *Ctla4*, and *Tgfb* were upregulated upon treatment with resveratrol, where increased *Foxp3* expression was attributed to upregulated aryl hydrocarbon receptor (AhR), which translocates to the nucleus and induces expression of Treg-related genes [39]. Interestingly, resveratrol showed a decrease in mRNA expression of the IL-17 receptor (*Il17r*) [39], which is a pro-inflammatory marker in various inflammatory diseases and age-related decline [124]. The decrease in *Il17r* expression and the increase in Tregs are likely connected, as Th17 and Treg cells are known to be reciprocally regulated [125]. Further research is warranted to evaluate whether resveratrol influences T cell fate during T cell development to promote Treg expansion and suppress IL-17 signaling. Another study showed similar findings, where resveratrol-treated obese mice increased the CD3+ CD4+/CD3+CD8+ ratio and anti-inflammatory CD4+CD25+FOXP3+ Treg production, along with a decrease in splenic mRNA expression of inflammatory markers such as *Rela* (p65 subunit of NF-κB), *Ptgs2* (COX-2), and *Tnf* [126]. Overall, these findings demonstrate that grape-derived resveratrol may have the ability to inhibit obesity-induced suppression of Tregs, while promoting a shift from pro-inflammatory Th1 to anti-inflammatory Th2 phenotypes [39].

### 4.2. Effects of Grapes and Grape-Derived Bioactives on Inflammation and Immune Profiles Within the Context of Hepatic and Adipose Tissue Dysfunction and Insulin Resistance in Experimental Models of Cardiometabolic Disease

Cardiometabolic diseases are often characterized by monocyte/macrophage infiltration and activation into metabolic tissues, leading to induction of pro-inflammatory mediators that exacerbate systemic inflammation, metabolic dysfunction, and insulin resistance [127,128]. A well-established mechanism involves the role of TNF-α in impairing insulin signaling by phosphorylation of serine residue 307 of IRS-1, thereby interfering with PI3K signaling pathway, leading to cardiometabolic diseases like diabetes and obesity [129]. In a study by Chuang et al. [36], grape powder extract mitigated TNF-α-induced inflammation and insulin resistance in human adipocytes. Accordingly, grape powder extract attenuated TNF-α-mediated activation of mitogen-activated protein kinase (MAPK), AP-1, and NF-κB activity, which in turn decreased TNF-α-mediated expression of inflammatory cytokines such as *IL6*, *IL1B*, *CXCL8* (IL-8), *CCL2* (MCP-1), *PTGS2* (COX-2), and *TLR2*, affecting the activation of the inflammatory cascade [36]. This further resulted in reduction of protein tyrosine phosphatase 1B (PTP-1B) and *p*-Ser307-IRS-1, negative regulators of insulin sensitivity in human adipocytes [36].

The regulation of inflammation and insulin receptor signaling by grape-derived compounds has additionally been demonstrated in vivo. In a high-fat-fructose-fed (HFF) Wistar rat model of metabolic syndrome, grape pomace and grape pomace extract supplementation attenuated *p*-Ser307-IRS-1 and c-Jun *N*-terminal kinase (JNK) activation, NADPH oxidase activity, and resistin expression in adipose tissue, while increasing phosphorylated-AKT and extracellular signal-regulated kinases (ERK) activity and the anti-inflammatory marker adiponectin—effects which were associated with decreased hepatic triacylglycerol accumulation and inflammation [130]. Similar findings were observed in another in vivo study conducted in HFF mice, which showed a reduction in mRNA expression of systemic inflammatory markers such as *Tnfa, Ifng, Il12b*, and the adipokine resistin expression following grape pomace extract supplementation [131]. Taken together, these findings indicate that grape pomace and grape pomace extract can improve insulin sensitivity and attenuate inflammation in adipose, hepatic, and systemic tissues [130,131].

Several studies have shown that grape-derived bioactives may exert cardiometabolic benefits through activation of sirtuin 1 (SIRT1), a nicotinamide adenine dinucleotide (NAD)+-dependent deacetylase enzyme, which plays a protective role in a number of metabolic pathways by modulating hepatic fat accumulation, glucose metabolism, and inflammation [132]. Findings from an in vitro study showed that grape seed proanthocyanidin extract restored intracellular triglycerides to the basal level and attenuated markers of hepatic steatosis in fat-overloaded HepG2 cells [37]. Grape seed proanthocyanidin extract-treated HepG2 cells also showed reduced mRNA expression of *FAS* and *ACC*, key genes involved in de novo lipogenesis. These protective effects were attenuated following pharmacological inhibition of SIRT1, suggesting involvement of the SIRT1 pathway in grape seed proanthocyanidin extract-mediated regulation of hepatic lipid accumulation [37]. Supporting this mechanism, the same study demonstrated that dietary grape seed proanthocyanidins increased hepatic NAD+ availability and *Sirt1* expression and its activity in healthy rats in a dose-dependent manner [37]. NAD+ bioavailability has been identified as one of the key determinants of SIRT1 activity, and higher NAD+ levels are related to higher SIRT1 enzymatic rates [133]. Aragonès et al. [37], demonstrated both of these effects simultaneously, suggesting that grape proanthocyanidins may enhance SIRT1 function not only by upregulating its expression but also by improving intracellular conditions required for its activation and function. Notably, resveratrol, another grape-derived polyphenol, has also been identified as a compound capable of activating SIRT1 [129] via additional mechanisms reviewed in [133], suggesting that multiple grape-derived bioactives may have an effect on SIRT1 activation through multiple complementary pathways.

While grape seed proanthocyanidins appear to enhance SIRT1 activity by increasing NAD+ availability, resveratrol has similarly been shown to regulate the SIRT1 pathway, resulting in attenuation of inflammation under hyperglycemic conditions. Accordingly, a recent study conducted by Tshivhase et al. [134] showed that resveratrol attenuated high glucose-induced expression of NF-κB pathway mediators in HepG2 cells, in addition to lowering mRNA expression of pro-inflammatory mediators *TNF*, *IL6*, *IL1B*, and *PTGS2* (COX2), while increasing expression of *SIRT1*. Similarly, SIRT1-mediated anti-inflammatory effects have been observed with other grape-derived products beyond resveratrol. In human endothelial cell line EA.hy926 cultured in hyperglycemic conditions, wine pomace fractions decreased NF-κB expression and activation, as evidenced by reduced ratios of phosphorylated inhibitor of κB kinase to inhibitor of κB kinase (pIKK/IKK) and phosphorylated inhibitor of κB alpha to inhibitor of κB alpha (pIκBα/IκBα), in addition to reducing mRNA expression of *PTGS2* (COX2) and *NOX4*. The researchers attributed this reduction to upregulated *SIRT1* expression [38].

Resveratrol and other grape-derived preparations have similarly been shown to mediate anti-inflammatory and protective metabolic effects in vivo. In HFD-fed C57BL/6 mice, resveratrol decreased both mRNA and protein levels of F4/80+ M1 macrophages and C-C motif chemokine receptor 2 (CCR2) levels, as well as mRNA expression of *Ccr2, Ccl2* (MCP-1) and *Tnf* in visceral adipose tissue [135]. Furthermore, Gao et al. [136] demonstrated that feeding 8-week-old Sprague-Dawley rats a HFD and resveratrol (200 mg/kg/day) for 6 weeks induced activation of the SIRT1 pathway, attenuated pro-inflammatory cytokines and NF-κB activity, and decreased the proportion of M1 macrophages, characterized by CD45+F4/80+CD11c+ cells, relative to M2 macrophages, characterized by CD45+F4/80+CD206+ cells, in epididymal white adipose tissue (eWAT). Findings from another in vivo study conducted in HFD-induced obese mice [137] similarly showed that grape seed oil supplementation reduced mRNA expression of pro-inflammatory macrophage markers such as *Adgre1* (F4/80) and *Nos2* (iNOS) and promoted macrophage polarization to the anti-inflammatory M2 phenotype characterized by an increase in *Mrc1* and *Arg1* expression. *Mrc1* encodes CD206, a hallmark marker of M2 macrophages which are generally associated with anti-inflammatory functions [138]. Macrophage reprogramming was associated with reduced serum levels and adipose tissue mRNA expression of pro-inflammatory markers such as TNF-α, IL-1β, and IL-6, thereby mitigating systemic inflammation and adipose tissue dysfunction [137]. Across these studies, M2 polarization emerges as a functional outcome of grape-derived interventions irrespective of specific bioactive compound used (i.e., resveratrol or grape seed oil), with findings suggesting that grape polyphenols as a whole may broadly promote anti-inflammatory macrophage phenotypes in adipose tissue. Another study conducted in C57BL/6 mice fed with HFD and grape seed proanthocyanidin extract exhibited lower levels of plasma TNF-α and IL-6 [42]. Further, mRNA expression of *Ccl2* (MCP-1) and macrophage markers *Adgre1* (F4/80) and *Cd68* were attenuated in both eWAT and the liver of grape seed proanthocyanidin extract-treated mice, showing decreased macrophage accumulation in both tissues [42]. Additionally, the study showed that grape seed proanthocyanidin extract ameliorated inflammation by inhibiting JNK and NF-κB signaling pathways and improved insulin sensitivity by increasing IkB-α and phosphorylated AKT levels in an HFD-fed mouse model [42]. Overall, these findings suggest that resveratrol and grape-derived oils and extracts ameliorate obesity-induced inflammation in adipose and hepatic tissues by reducing monocyte/macrophage infiltration and modulating macrophage polarization to the anti-inflammatory M2 phenotype, which is associated with improved insulin sensitivity.

The significance of immune cell activation, phenotype, and secretomes in modulating adipose tissue function is supported by research studies evaluating ex vivo adipose tissue responses to macrophage-conditioned media following treatment with grape-derived bioactives. Accordingly, two separate studies demonstrated that conditioned media from LPS-activated macrophages, generated in the presence of grape powder extract and the grape-derived bioactive, quercetin, reduced inflammation and improved insulin signaling in human adipocytes derived from abdominal white adipose tissue after abdominoplasty [139,140]. When human adipocytes were treated with conditioned media from macrophages, grape powder extract attenuated mRNA expression of LPS-mediated pro-inflammatory markers such as *IL6*, *CXCL8* (IL-8), *CCL2* (MCP-1), and *IL1B*, decreased activation of NF-κB, and decreased the impairment of insulin-stimulated glucose uptake [139]. Similarly, when human adipocytes were incubated with conditioned media from macrophage pre-treated with quercetin, inflammation was decreased, as evidenced by reduced mRNA expression of *TNF*, *CCL2* (MCP-1), *CXCL10* (IP-10), and *PTPN1* (PTP1B) expression, as well as decreased phosphorylation of cJUN and JNK and *p*-Ser307-IRS-1 [140]. These studies demonstrate that grape powder extract and quercetin reduced the inflammatory capacity of macrophages, the macrophage secretome, and their ability to induce insulin resistance and inflammation.

### 4.3. Effects of Grapes and Grape-Derived Bioactives on Inflammation and Immune Profiles Within the Context of Cardiovascular Disease, Hypertension, and Vascular Function

The regulation of inflammatory and immune-mediated mechanisms by grape and grape-derived bioactives has also been shown to exert atheroprotective and anti-hypertensive effects. In an in vivo study, Zhou et al. [141] showed that proliferation and activation of CD4+ T cells in ApoE-/- mice subjected to HFD+LPS-induced atherosclerosis were increased, which led to atherosclerotic plaque formation and increased secretion of inflammatory markers and elevated expression of DNA methyltransferases. However, resveratrol treatment attenuated atherosclerosis induced by HFD+LPS in ApoE-/- mice by inhibiting the proliferation and activation of CD4+ T cells, as characterized by a reduction in Ki-67 (CD4+ T cell proliferating marker), CD25, and CD44 activation markers, and shifted the cytokine profile towards an anti-inflammatory phenotype by reducing IL-6 and increasing IL-10 and transforming growth factor-beta (TGF-β) secretion [141]. The immunomodulatory effects may in part be due to resveratrol-mediated epigenetic effects, as DNA methyltransferases, Dnmt1 and Dnmt3b—which maintain DNA methylation and mediate de novo methylation, respectively, and are best characterized for epigenetic modifications—were also shown to be decreased with resveratrol treatment. Overall, the study concluded that resveratrol was able to reduce atherosclerotic burden, which was attributable to modulation of CD4+ T cell activity [141]. This CD4+ T cell suppression in atherosclerosis parallels the Treg restoration observed in obesity models [39,126], which suggests that resveratrol modulates T cell activity across different disease contexts and shifts the immune balance toward an anti-inflammatory phenotype.

Beyond atherosclerosis, the benefits of grape powders and oils have been observed in different CVD models. Seymour et al. [142] reported that a 5-week-old rat model of salt-sensitive hypertension and diastolic dysfunction was fed with low-salt or high-salt diets with or without freeze-dried whole grape powder for 18 weeks. Whole grape powder-fed rats exhibited increased DNA binding activity of PPARs, such as PPARα and PPARγ, and decreased transcriptional activity of NF-κB [142]. This was accompanied by increased mRNA expression for *Ppara*, *Pparg*, and peroxisome proliferator-activated receptor gamma coactivator 1-alpha (PGC-1α, *Ppargc1a*), supporting the transcriptional PPAR activity. Whole grape powder-fed rats also showed increased mRNA expression of *Nfkbia* (Ikba) and decreased mRNA of multiple pro-inflammatory cytokines such as *Tnf*, *Il6*, growth factors such as *Tgfb*, and adhesion molecules such as *Icam1*, supporting the reduced NF-κB activity [142]. They also showed reduced cardiac TNF-α and TGF-β levels, further attenuating cardiac-related inflammation [142]. In a rat model of isoproterenol (ISO)-induced acute myocardial infarction (MI), pretreatment with grape seed oil for 14 days resulted in a significant decrease in inflammation characterized by a decrease in IL-6, IL-1β, and TNF-α levels, suggesting an anti-inflammatory effect in ischemic heart conditions [143]. Similar results were found in another study conducted by Pop et al. [144] which used higher and lower doses of whole grape pomace in ISO-induced MI rats. The authors observed that serum and cardiac tissue protein levels of pro-inflammatory cytokines such as TNF-α and IL-1β were reduced. In contrast, whole grape pomace exerted only modest effects on IL-6 protein levels, with no significant differences observed between treatment groups within cardiac tissue [144]. The authors suggested that these anti-inflammatory effects may be associated with modulation of NF-κB-mediated inflammatory signaling pathways, based on previous studies demonstrating inhibitory effects of grape pomace extracts on NF-κB activation [144].

Together, these findings demonstrate that whole grape preparations and specific grape-derived bioactives (i.e., resveratrol) exert anti-inflammatory and immunomodulatory effects that protect against CVD and vascular dysfunction in animal models. These effects appear to be related to a common mechanism: suppression of the NF-κB pathway, which is linked to PPARα/γ activation, modulation of CD4+ T cell activity, and reduction of pro-inflammatory cytokine expression, suggesting that regulation of the NF-κB pathway may be a central target underlying the cardioprotective effects of grapes.

### 4.4. Effects of Grapes and Grape-Derived Bioactives on Inflammation and Immune Profiles Within the Context of Aging and Senescence

In line with beneficial anti-inflammatory and immunomodulatory effects within the context of cardiometabolic disease, grapes and grape-derived compounds are known to exert beneficial effects on markers of aging and senescence. Findings from both in vitro and in vivo experiments showed that grape seed extract and procyanidin C1, a bioactive found in grapes, alleviated cellular senescence and its associated inflammation [40]. Grape seed extract and procyanidin C1 showed the ability to clear senescent PSC27 cells, and procyanidin C1 further showed senolytic activity across various cell lineages, such as human stromal cells, human fetal lung fibroblasts, primary human umbilical vein endothelial cells (HUVECs), and human mesenchymal stem cells (MSCs) [40]. RNA-seq data showed that grape seed extract treatment decreased molecular signatures of SASP and activation of NF-κB, a key mediator of inflammation [40]. Procyanidin C1 decreased p53 nuclear translocation and increased its translocation to mitochondria. P53 in the mitochondria binds to pro-apoptotic proteins like BCL2-associated X protein (BAX), which triggers the release of cytochrome C. This further activates caspase-3, which induces apoptosis in senescent cells [40]. Further, Procyanidin C1-treated aged mice showed a depletion in senescent cells across multiple tissues, and Gene Set Enrichment Analysis (GSEA), a method that evaluates whether predefined gene sets are collectively upregulated or downregulated [145], showed decreased SASP-associated genes and inflammation [40]. Another in vitro study showed that resveratrol treatment in aged vascular smooth muscle cells (VSMCs) derived from the non-human primate *Macaca mulatta* reversed age-related changes [146]. Notably, resveratrol decreased the secretion of pro-inflammatory cytokines such as IL-1β, IL-8, TNF-α, and MCP-1 in aged VSMCs [146]. Similarly, in an in vivo study, 24-month-old C57BL/6 mice were fed a HFD to induce metabolic syndrome. This experimental mouse model, when treated with resveratrol, also showed a decrease in pro-inflammatory cytokines such as TNF-α, IL-1, and IL-6, which was attributed to modulation of SIRT1-AMPK-PGC-1α cellular pathways [147]. Findings from both in vitro and in vivo experiments showed that grape seed proanthocyanidin extract-supplemented aged C57BL/6 mice and grape seed proanthocyanidin extract-treated aging retinal pigment epithelial cells (RPEs) exhibited an upregulation of nicotinamide phosphoribosyltransferase (NAMPT) and intracellular NAD+, both of which are key regulators in aging [46]. Grape seed proanthocyanidin extract-supplemented mice showed a decrease in cellular senescence-related markers such as p16 and p21 and mRNA expression of SASP-associated factors such as *Il18*, *Cxcl15* (IL-8), *Timp2*, *Mmp3*, *Ccl2* (MCP-1), and others [46]. Furthermore, grape seed proanthocyanidin extract-treated RPE cells showed a decrease in β-gal activity, an increase in barrier function markers such as ZO-1 and transepithelial/transendothelial electrical resistance (TEER), and attenuated SASP-associated factors. These cells also reduced NLRP3 expression, attenuating inflammasome activity, and attributed this decrease to increased SIRT1 activity [46]. Altogether, grape seed proanthocyanidin extract may decrease cellular senescence in age-related degenerative disorders via the NAMPT/SIRT1/NLRP3 pathway [46].

### 4.5. Antioxidant Activity of Grapes in the Regulation of Inflammation in Cardiometabolic Diseases and Aging

It is well established that oxidative stress plays a central role in chronic low-grade inflammation, immune dysregulation, and the pathogenesis of cardiometabolic and age-related diseases [148,149]. The excessive accumulation of ROS and reactive nitrogen species (RNS), primarily generated by NADPH oxidase, xanthine oxidase, and nitric oxide synthase (NOS), triggers redox-sensitive signaling cascades such as NF-κB, AP-1, and MAPK pathways [150]. This activation induces the transcription of pro-inflammatory cytokines (TNF-α, IL-1β, IL-6), adhesion molecules (VCAM-1, ICAM-1), and enzymes, including cyclooxygenase-2 (COX-2) and inducible nitric oxide synthase (iNOS). Collectively, these processes increase oxidative and inflammatory tissue injury in vascular, hepatic, and adipose compartments and contribute to inflammaging [150]. Grape-derived polyphenols including anthocyanins, proanthocyanidins, catechins, and stilbenes such as resveratrol, exhibit potent antioxidant activity [13,14]. Grape compounds can directly scavenge reactive oxygen and nitrogen species (ROS and RNS) while also inhibiting pro-oxidant enzymatic systems like NADPH oxidase, thereby lowering primary oxidative damage. In addition, grape compounds activate natural antioxidant defense systems via Nrf2/heme oxygenase-1 (HO-1) and SIRT1-mediated pathways, increasing the expression and activity of antioxidant-related enzymes such as superoxide dismutase (SOD), catalase (CAT), and glutathione peroxidase (GPX) [14,150]. Together, these likely mediate, in part, the anti-inflammatory, immunomodulatory, and health-promoting properties of grapes. The following sections highlight research characterizing the antioxidant properties of grape and grape-derived compounds in regulating inflammation and immune pathways in cardiometabolic disease and aging models.

#### 4.5.1. Regulation of Inflammation and Antioxidant Activity by Grapes in Models of Obesity and Insulin Resistance

In an in vivo study, male Sprague-Dawley rats were fed a HFD with or without 5% grape seed supplementation [151]. Grape seed-supplemented rats showed reduced serum and hepatic levels of lipid peroxidases compared to non-grape seed-fed rats [151]. There was also an upregulation in antioxidant enzyme activities such as SOD, CAT, GPX, and glutathione-S-transferase in grape seed-fed rats, which are efficient in protecting the cells and tissues from oxidative stress. Moreover, the glutathione (GSH) to glutathione disulfide (GSSG) ratio was increased after supplementing with the grape seed diet [151]. The GSH/GSSG ratio reflects the cellular redox status: a high ratio indicates abundant reduced glutathione and low oxidative stress, whereas a decreased ratio signifies glutathione depletion and increased oxidative burden [152], as commonly observed in conditions such as obesity, hyperglycemia and cardiovascular disease where oxidative stress plays a complex role throughout their occurrence and progression [153,154]. Another in vivo study showed that C57BL/6 mice were fed a high-fat diet to induce obesity and then supplemented with resveratrol for 13 more weeks [126]. The study showed that HFD-induced obesity decreased splenic CAT activity, total antioxidant capacity, GPX, and GSH/GSSG ratio and increased MDA levels. However, resveratrol-supplemented mice showed an upregulation in CAT activity and GSH/GSSG ratio and decreased MDA levels, thereby decreasing oxidative stress induced by obesity [126]. These studies together show that HFD-induced obesity depletes endogenous antioxidant defenses, and grape-derived compounds appear to partially restore them via different mechanisms, highlighting the role of grape bioactives in dysregulated redox environments in metabolically stressed conditions.

Grape-mediated antioxidant effects may be directly protective against hyperglycemia-induced inflammation. In renal epithelial cells under hyperglycemic stress, grape seed polyphenols attenuated NF-κB activation and reduced COX-2 and iNOS protein expression, highlighting their potential to mitigate hyperglycemia-associated oxidative injury relevant to diabetic cardiometabolic pathology [155]. Findings from another in vitro study showed that hyperglycemia-induced endothelial cells showed significantly reduced *NFE2L*2 (encoding NRF2) mRNA expression and decreased phosphorylated nuclear factor erythroid 2-related factor 2 (NRF2)/NRF2 ratio [38]. Nrf2 is a master transcription factor that regulates the expression of antioxidant related genes, and its activation enhances cellular defenses against oxidative stress and inflammation [156]. Phosphorylation of Nrf2 promotes its activation and promotes its release from inhibitory proteins, nuclear translocation, and subsequent induction of antioxidant gene expression [157]. Treatment with wine pomace fractions restored these levels, alongside increased mRNA expression of glutathione metabolism enzymes and the GSH/GSSG ratio—a marker of cellular redox status [38]. Further, wine pomace fraction after colonic fermentation (WPF) exhibited broader antioxidant enzyme upregulation than wine pomace fraction after gastrointestinal digestion (WPGI), increasing *SOD1*, *CAT*, *GPX1*, *HMOX1* (HO-1), and *NQO1* mRNA expression, while WPGI only significantly increased *CAT*, *HMOX1* (HO-1), and *NQO1* [38]. This difference is likely due to greater bioactivity of compounds generated during colonic fermentation compared to those absorbed during gastrointestinal digestion. However, both fractions reduced pro-oxidant enzyme *PTGS2* (COX-2) and *NOX4* mRNA expression, consistent with reduced NF-κB activity, which led to the reduction in oxidative stress and inflammation in hyperglycemic cells [38].

#### 4.5.2. Regulation of Inflammation and Antioxidant Activity by Grapes in Models of CVD

Whole grape preparations and specific grape-derived bioactives have shown antioxidant and disease outcome benefits related to MI, vascular remodeling, and hypertension. A recent study conducted by Pop et al. [144] in ISO-induced MI rats showed that MI induction led to an increase in pro-oxidant parameters such as total oxidative stress, nitric oxide (NO), and MDA, a key marker of lipid peroxidation, and showed a higher oxidative stress index, which usually activates the iNOS pathway, leading to greater oxidative stress. Upon treatment with whole grape pomace for 14 days, there was a reduction in all these parameters, leading to a decrease in oxidative stress [144]. Furthermore, malvidin, a bioactive component found in grapes, showed an antioxidant effect in MI-induced rats [43]. This experimental rat model, when treated with malvidin, showed reduced lipid peroxidation, as evidenced by lower MDA levels inhibiting oxidative damage to the cardiac tissue [43]. These rats also showed greater myocardial antioxidant enzyme activities (CAT, SOD), and elevated GSH levels, alongside increased expression of transcription factor Nrf-2 and HO-1, providing cardioprotective effects via the Nrf2/HO-1 activation pathway [43]. The study also demonstrated that malvidin treatment not only showed antioxidant but also anti-inflammatory properties as evidenced by inhibition of pIkB-α and NF-κB activation and reduced serum pro-inflammatory markers such as IL-6 and TNF-α [43]. Nrf2/HO-1 activation and NF-κB suppression are known to be reciprocally regulated. Under oxidative stress, the Nrf2 protein detaches from Keap-1, migrates to the nucleus, and triggers the transcription of cytoprotective enzymes such as HO-1 [158]. HO-1 and its byproducts inhibit the degradation of IκBα thereby suppressing NF-κB activity [153], suggesting malvidin’s anti-inflammatory effects within the cardiac context may be downstream of antioxidant signaling.

Similar effects have been observed with resveratrol. In an in vitro study by Movahed et al. [159], adult cardiomyocytes exposed to hydrogen peroxide (H_2_O_2_) exhibited higher amounts of oxidative stress and reduced antioxidant enzyme activities. This effect was prevented when these cells were pretreated with resveratrol, which decreased oxidative stress measured by lactate dehydrogenase activity and increased antioxidant-related enzyme activities such as SOD and CAT in H_2_O_2_-exposed cardiomyocytes [159]. These results were consistent with in vivo experiments, where 10-week-old adult spontaneously hypertensive rats (SHR) showed decreased H_2_O_2_ levels and increased antioxidant-related enzymes such as CAT after treatment with resveratrol [159].

Antioxidant benefits of grape compounds are consistently observed in hypertension models. An in vivo study conducted by Wang et al. [160] showed that when hypertension-induced deoxycorticosterone acetate (DOCA)-salt mice were treated with oligomeric grape seed proanthocyanidin for 4 weeks, there was an amelioration of endothelial dysfunction, which was attributed to an increase in NO production and decreased MDA levels. This study also showed that grape seed proanthocyanidin treatment improved cardiac remodeling, highlighting a decrease in oxidative stress and enhancing vascular antioxidant defenses [160]. Similarly, an earlier study showed that in Sprague-Dawley rats fed a high-fructose diet, grape skin extract reduced cardiac ROS production by downregulating NADPH oxidase, which mitigated hypertrophic signaling and protected against cardiac remodeling and hypertension [161]. The suppression of NADPH oxidase by grape skin extract is notable as NADPH oxidase is a primary source of ROS in vascular tissue and is implicated in conditions such as hypertension, where excessive ROS production drives vascular remodeling and endothelial dysfunction [162]. Red wine pomace supplementation has additionally been shown to have anti-hypertensive and antioxidant properties when supplemented in 12-week-old spontaneously hypertensive rats [163]. Red wine pomace-supplemented rats showed a reduction in systolic blood pressure (SBP) and decreased markers of oxidative stress, such as plasma MDA levels, and increased endothelial NO production and bioavailability [163]. These rats also showed an increase in aortic mRNA expression of endothelial function and antioxidant-related markers such as *Nos3*, *Sod2*, and *Hmox1* (HO-1) and reduced *Ace* expression. However, this study showed no effect on *Nox4* expression [163]. In contrast, wine pomace fractions reduced *NOX4* mRNA expression in hyperglycemic endothelial cells [38], suggesting that the effect of grape-derived compounds on NADPH oxidase may vary depending on the disease context or specific preparation or the model used.

Similarly, resveratrol and its oligomers (ε-viniferin, δ-viniferin) enhanced endothelial NO production via endothelial NOS (eNOS) phosphorylation and SIRT1/HO-1 upregulation, while increasing CAT expression and protecting vascular endothelial cells from H_2_O_2_-induced oxidative injury [164]. Pharmacological inhibition of eNOS, HO-1, or SIRT1 abolished these effects, highlighting their mechanistic indispensability in vascular redox homeostasis [164]. Together, these findings demonstrate that grape-derived compounds protect against cardiovascular oxidative stress through multiple mechanisms such as scavenging ROS, suppressing pro-oxidant enzymes, and inducing endogenous antioxidant pathways; however, the consistency of these effects varies across different grape-derived compounds, disease models, and tissue types.

#### 4.5.3. Regulation of Inflammation and Antioxidant Activity by Grapes in Models of Aging

As detailed above, increased oxidative stress is a hallmark of aging. Aging is associated with a progressive decline in endogenous antioxidant capacity, characterized by reduced Nrf2 responsiveness, depleted glutathione and accumulated oxidative damage, contributing to inflammatory phenotype [165]. However, NO, which is normally protective in nature, can be excessively produced during aging, contributing to RNS-mediated oxidative stress [165]. In cardiomyocytes isolated from heart tissue of aging rats treated with resveratrol for 2 to 8 months, there was a reduction in NO levels, indicating lower oxidative stress [166]. Additionally, lipid peroxide products such as MDA and MDA+4-hydroxyalkenals (4-HDA) levels were lower, causing less peroxidation, thereby decreasing oxidative stress. However, this study did not see any significant changes in antioxidant enzyme activities and attributed decreased oxidative stress to direct scavenging activity of resveratrol [166]. In contrast to this study, evidence from aged non-human primate vascular smooth muscle cells showed that resveratrol activated Nrf2 transcriptional activity in a dose-dependent manner alongside significant reductions in mitochondrial superoxide production and suppression of NF-κB activity [146], even though it is compromised in other cell types [166]. While Csiszar et al. [146] demonstrated Nrf2 activation and antioxidant responses in aged vascular tissue, evidence from metabolic tissues suggests that grape-derived compounds can similarly restore antioxidant responses in other aging models. In D-galactose-induced aging mice, grape seed proanthocyanidin extract reduced hepatic and colonic MDA and MPO activity while increasing SOD and GPX levels, along with suppression of TNF-α and IL-1β and inhibition of NLRP3 inflammasome [44]. Similarly, in aged female Wistar rats, grape seed proanthocyanidin extract reduced MPO activity specifically in jejunum alongside improvements in gut barrier function [45]. A study by Fernandes et al. [167] investigated resveratrol effects on antioxidant and inflammatory gene expression in peripheral blood mononuclear cells (PBMCs) from young and elderly female volunteers upon TLR4 and TLR7/8 stimulation. Resveratrol upregulated *CAT* expression in both age groups and *SIRT1* specifically in elderly cells, suggesting an age-dependent enhancement of antioxidant responses [167]. Resveratrol also reduced pro-inflammatory cytokines such as TNF-α and IFN-γ and chemokines CCL2 and CCL5 across both age groups and TLRs [167].

Taken together, findings across various experimental models and tissues suggest that grape-derived compounds, particularly resveratrol and grape seed proanthocyanidin extract, may have a broader capacity to restore antioxidant defenses that decline with aging. The mechanisms underlying these effects vary by tissue type, ranging from direct ROS scavenging in cardiac tissue to Nrf2 activation in vascular tissue and to enzymatic restoration in metabolic and gut tissues. Importantly, across all aging models, improvements in oxidative stress markers corresponded with reductions in inflammatory markers, which suggests that grape-derived antioxidant effects in aging may also be linked with their anti-inflammatory effects.

### 4.6. The Role of the Gut Microbiome in Mediating the Anti-Inflammatory and Immunomodulatory Effects of Grapes in Cardiometabolic Diseases and Aging

The gut microbiome plays a critical role in the pathophysiology of cardiometabolic disease and inflammaging [168]. Accordingly, the anti-inflammatory and immunomodulatory effects of grapes and grape-derived compounds may in part be due to changes in the gut microbiome, whereas the composition of the gut microbiome may simultaneously influence the generation of grape-derived metabolites known to impact disease progression and longevity [169,170]. Because most grape polyphenols (such as anthocyanins, proanthocyanidins, and flavan-3-ols) and stilbenes (such as resveratrol) are largely resistant to absorption in the small intestine, they reach the colon largely intact, where they undergo microbial biotransformation [171]. Gut bacteria convert them into smaller metabolites such as phenolic acids, phenyl-γ-valerolactones, and urolithins through glycosidic bond cleavage, ring decarboxylation, and hydroxylation [170]. These metabolites exert systemic effects by modulating inflammatory pathways, including NF-κB signaling and cytokine production, reducing endothelial adhesion molecule expression, and enhancing antioxidant defenses, thereby influencing vascular and endothelial function [172]. The following section outlines the effects of grapes and grape-derived compounds on gut microbiome profiles in models of cardiometabolic disease and aging.

#### 4.6.1. Gut Microbiome Modulation by Grapes in Models of Obesity and Insulin Resistance

Several studies have demonstrated that grape polyphenols can partially restore gut dysbiosis induced by obesogenic diets, with consistent enrichment of beneficial taxa and suppression of pro-inflammatory species. Han et al. [173] showed that grape extract supplementation (1% *w*/*w*) in high-fat and high-fructose diet (HFFD)-induced obese mice normalized gut dysbiosis and increased the abundance of *Verrucomicrobia*, *Bifidobacterium*, and *Akkermansia*, while reducing *Bacteroides* and *Desulfovibrio*. These microbial shifts were accompanied by changes in the bile acid pool, specifically increased secondary bile acids that activate the Takeda G-protein-coupled receptor 5 (TGR5) in brown adipose tissue, improving thermogenesis and glucose tolerance and adiposity [173]. Importantly, grape extract metabolites did not activate TGR5 in vitro, confirming that these metabolic benefits are entirely dependent on microbiome-mediated bile acid transformation, further highlighting the gut microbiome as an essential mediator of grape polyphenols. Similarly, grape seed proanthocyanidin extract supplementation in HFD-fed mice increased butyrate-producing bacterial species such as *Clostridium XIVa, Roseburia spp*. and *Roseburia inulinivorans* [42]. It is interesting to note that certain Clostridia clusters, such as XIVa, have been shown to activate Treg cells, modulate host fatty acid metabolism, and attenuate host colitis, and butyrate-producing bacteria are considered to be probiotics in certain intestinal diseases that help maintain immune homeostasis [174,175]. Notably, grape seed proanthocyanidin extract also decreased *Firmicutes* and increased abundance of *Proteobacteria* in this model [42].

Beyond microbiota composition, grape polyphenols consistently improve intestinal barrier integrity in obesity models, which is a functionally important outcome because a compromised gut barrier allows bacterial endotoxins such as LPS to enter systemic circulation and trigger inflammatory cascades [176]. Grape seed proanthocyanidin extract supplementation in cafeteria diet-fed Wistar rats significantly reduced intestinal permeability and normalized MPO activity, which is a key intestinal inflammatory marker and an index of neutrophil infiltration [177]. Grape seed proanthocyanidin extract-supplemented rats also showed reduced LPS and TNF-α levels and increased claudin-1 gene expression and showed small changes in tight junction proteins. Overall, grape seed proanthocyanidin extract protected against intestinal barrier dysfunction and attenuated intestinal and systemic inflammation in a rat model of obesity [177]. Similarly, Roopchand et al. [178] demonstrated that dietary Concord grape polyphenols markedly increased *Akkermansia muciniphila* and lowered the Firmicutes-to-Bacteroidetes ratio in HFD-fed mice, correlating with enhanced intestinal barrier function attributed to upregulation of tight junction proteins such as occludin, reduced intestinal inflammation, and downregulated glucose absorption genes. Across these studies that used different grape compounds, such as resveratrol, grape extract, and Concord grapes, there was a consistent enrichment of *Akkermansia muciniphila*, which may be a mechanistic target for grape compounds as a whole. Because many grape polyphenols are poorly absorbed and reach the colon largely intact, they may alter the gut environment, which may favor *Akkermansia* growth, although the precise mechanisms remain incompletely understood [178,179].

#### 4.6.2. Gut Microbiome Modulation by Grapes in Models of Cardiovascular Disease

Gut dysbiosis has been directly implicated in atherosclerosis progression, partly through elevated circulating trimethylamine-*N*-oxide (TMAO), a metabolite generated from gut microbiota-derived trimethylamine (TMA), which is associated with foam cell formation and vascular inflammation [180]. Gram-negative bacteria release LPS upon gut barrier disruption, further amplifying this inflammatory cascade by binding to TLR4 on macrophages and endothelial cells, promoting systemic low-grade inflammation and progression of atherosclerosis [180]. Grape-derived compounds have shown potential to attenuate atherosclerosis by targeting these mechanisms, reshaping the gut microbiome, reducing TMAO production, and improving gut barrier integrity. Chen et al. [41] demonstrated that resveratrol attenuated TMAO in ApoE-/- mice by increasing bacteria with high bile salt hydrolase activity, such as *Bacteroides*, *Lactobacillus*, *Bifidobacterium*, and *Akkermansia*, while suppressing *Prevotella* and *Ruminococcaceae*. This shift in gut microbiota composition promoted bile acid deconjugation and fecal excretion, suppressing TMA production and consequently reducing TMAO levels, thereby attenuating atherosclerosis progression [41]. These findings suggest that the atheroprotective effects of resveratrol may be partly mediated through gut microbial derivatives, as the concurrent shifts in microbial composition and TMAO levels may be mechanistic links between resveratrol consumption and cardiovascular protection.

Similar findings were observed in an ApoE-/- atherosclerosis model using Cabernet Sauvignon dry red wine, a whole red wine preparation containing a complex mixture of polyphenols [181]. Red wine supplementation significantly reduced Firmicutes/Bacteroidetes ratio, which is a widely used marker of gut dysbiosis, and increased the abundance of *Akkermansia* and *Muribaculaceae* while suppressing pro-inflammatory taxa. Notably, an increase in *Akkermansia* abundance negatively correlated with atherosclerotic plaque percentage, LDL-C, and inflammatory cytokines IL-1β and IL-6, and it positively correlated with HDL-C and eNOS levels, suggesting that *Akkermansia* enrichment may directly contribute to the cardiovascular benefits observed [181]. Interestingly, the low dose of red wine showed greater *Akkermansia* enrichment than the high dose, suggesting a non-linear dose–response relationship that warrants further investigation [181].

Together, these findings suggest the anti-inflammatory and atheroprotective effects of different grape-derived preparations and polyphenols may in part be attributable to restoration of gut microbiome profiles, with *Akkermansia* enrichment emerging as a consistent feature across studies in cardiovascular disease models.

#### 4.6.3. Gut Microbiome Modulation by Grapes in Models of Aging

Age-related dysbiosis is characterized by decreased microbial diversity; loss of beneficial bacteria such as *Bifidobacterium*, *Clostridiales* and *Akkermansia*; enrichment of pro-inflammatory pathobionts such as *Enterobacteriaceae* and *Desulfovibrio*; and decreased ratios of Firmicutes/Bacteroidetes, all of which contribute to intestinal barrier dysfunction and systemic inflammation in aging models [107,182]. Grape seed extract rich in proanthocyanidin supplementation in aged female Wistar rats showed beneficial effects on gut microbiome and inflammatory markers [45]. Aged rats supplemented with grape seed extract rich in proanthocyanidin not only showed significant improvements in gut microbiome with increased abundance of *Clostridium*, *Bacteroides*, *Bifidobacterium*, *Prevotella*, and *Akkermansia* but also recovered lost strains such as *Prevotella*, alongside reductions in pathobionts including *Rikenellaceae*, and *Desulfovibrio*. Overall, the proanthocyanidin-rich grape seed extract induced changes in the gut microbiome in aged rats to better resemble those observed in younger rats [45]. Further, grape seed extract also improved intestinal barrier function by increasing the mRNA expression of *Muc2*, a gene that maintains the mucus layer and protects gut permeability; it also decreased intestinal inflammation and neutrophil infiltration, as characterized by reduced MPO and LPS levels [45]. However, this study was conducted exclusively in female rats, and given that there are known sex differences in gut microbiota composition, whether these findings extend to males remains to be established [45].

Consistent with these findings, Sheng et al. [44] showed that grape seed proanthocyanidin extract in a D-galactose-induced aging mouse model increased the abundance of beneficial bacteria such as *Lactobacillus*, *Bifidobacterium* and *Akkermansia*, which are known to promote gut barrier integrity and reduce inflammation, while suppressing aging-associated pathobionts such as *Helicobacter* and *Alistipes* (species specifically linked to systemic inflammation and age-related disease). These microbial shifts were accompanied by restored expression of epithelial tight junction molecules such as zonula occludens 1 (ZO-1), occludin, claudin-1, along with downregulation of pro-inflammatory molecules [44]. Mechanistically, grape seed proanthocyanidin extract inhibited the NLRP3 inflammasome, a key marker of age-related inflammation, however, whether gut microbiome modulation mediates inflammatory suppression remains to be directly established [44].

Taken together, these findings suggest that grape polyphenols may be relevant in aging where gut dysbiosis actively drives inflammation. The consistent enrichment of gut beneficial bacteria such as *Akkermansia*, *Bifidobacterium* and SCFA-producing bacteria across different aging models, along with improvements in gut barrier function and inflammation, makes grape polyphenol-mediated microbiome modulation a promising strategy for attenuating inflammaging.

## 5. Bioactive Properties of Grapes and Grape Components Within the Framework of Cardiometabolic Health and Aging: An Update from Recent Intervention Trials

From the evidence presented above, it is clear that grape and grape components have the capacity to regulate inflammatory and immune-mediated mechanisms—both directly and via antioxidant and gut microbiome-mediated mechanisms—with relevance to cardiometabolic disease risk and healthy aging. Strong preclinical evidence has been supported to some degree by clinical trials; however, studies that specifically examine inflammatory and immune profiles and mechanisms in populations with varied cardiometabolic disease risk and age groups are overall lacking or have yielded neutral or minimal effects, and limited studies addressing these areas have been conducted within the last 5 years. That said, it is important to note that variability in study results may in part be attributable to differences in the health status of the study population, grape product preparation, and overall study design.

The effects of grapes and grape-derived products on inflammation, immune function, and cardiometabolic health markers have been investigated in human clinical trials for several decades [50]. Early evidence demonstrated that purple grape juice consumption inhibited platelet aggregation in healthy adults, an effect specific to grape flavonoids and not observed with other fruit juices, suggesting a cardiovascular protective role of grape polyphenols [183]. Subsequently, clinical trials have demonstrated that grape-derived products may modulate inflammatory and immune markers in populations with cardiometabolic risk [184,185,186]. Castilla et al. [184] reported reductions in oxLDL and MCP-1 in hemodialysis patients following red grape juice supplementation; reductions in TNF-α were observed following grape powder supplementation in pre-and postmenopausal women [185], and increases in anti-inflammatory IL-10 and adiponectin levels in plasma were found in men with metabolic syndrome without dyslipidemia [186]. The effects of grape-derived bioactives on cardiometabolic health and immune function have been previously reviewed [121,187,188]. Rasines-Perea and Teissedre [121] and others [189] reviewed grape and wine polyphenol effects on CVD and T2DM, concluding that grape polyphenols may support cardiovascular health through inhibition of platelet aggregation, reduced LDL oxidation, decreased oxidative stress, and favorable effects on blood lipids, decreased inflammation, and potentially reduced blood pressure, though results vary depending on grape product type (including confounding effects of alcohol in wine studies), dose and study population. Leifart and Abeywardena [187] similarly reviewed cardioprotective actions of grape polyphenols concluding that grape extracts and red wine contain bioactive components that offer some protection against CVD through multiple mechanisms supported by in vitro, animal, and limited human trial data. Petersen and Smith [122] reviewed grape-derived products specifically in the context of aging-associated oxidative stress and inflammation across multiple experimental models including limited human studies and concluded that grape-derived products consistently demonstrated beneficial effects on oxidative stress and inflammation, within the context of aging and age-associated conditions. However, comprehensive reviews of human clinical trial evidence specifically evaluating grape-derived bioactives in the context of aging are currently lacking.

More recently, Izadfar et al. [188] reviewed the effects of grapes and their products specifically on immune system function and concluded that grape-derived polyphenols may support immune function through an increase in lymphocyte activity, the phagocytic ability of macrophages, gamma-delta T cells, and antioxidant status. However, clinical trial evidence is limited, and findings are variable because of sample size, the population studied, dose and duration, and type of grape products. A systematic review and meta-analysis of 29 randomized controlled trials from inception to January 2022, most of which were published prior to 2020, examined the effects of whole grape products including grape juice, grape powder, raisins, currants, and pomace but excluding grape seed extract, resveratrol, and other isolated grape compounds [50]. The authors reported that grape products consistently improved antioxidant-related markers such as CAT, oxygen radical absorbance capacity (ORAC), and total antioxidant capacity (TAC) and reduced thiobarbituric acid reactive substances (TBARS), an indicator of lipid peroxidation. Grape products showed no significant effect on oxLDL, MDA and SOD. However, subgroup analysis revealed that whole grape extract supplementation significantly reduced oxLDL levels [50]. This meta-analysis also revealed that grape/whole grape products showed no significant overall effect on circulating inflammatory markers including hsCRP, IL-6, TNF-α, and MCP-1 in adults. However, subgroup analysis revealed that whole grape extract significantly reduced hsCRP and TNF-α compared to other types of intervention such as fresh grape or dried grape [50], which may suggest that the type of grape product influences antioxidant-related markers and inflammatory outcomes. It is important to note that this meta-analysis pooled diverse whole grape products across heterogenous populations including healthy adults, hemodialysis patients, and those with metabolic syndrome, diabetes, hypertension and other conditions; it excluded studies related to grape seed extract and isolated polyphenol compounds, both of which may have contributed to the non-significant overall inflammatory findings [50], and limited direct comparisons with trials using grape seed extract or resveratrol reviewed in the present paper.

Grape polyphenols are involved in regulating molecular pathways associated with the prevention of aging and their related muscle comorbidities in cell culture and animal studies, while human trials—particularly those investigating the effects of grapes in inflammaging, immune function, and immunosenescence—are scarce [190]. A recent study showed that dried fruit intake was associated with telomere length as determined by Mendelian randomization [191]. Telomeres are repetitive DNA sequences located at the ends of chromosomes that are bound by protective proteins that maintain chromosomal stability and can serve as a marker of biological aging that correlates with inflammaging and immunosenescence [192]. The “dried fruit” variable included one prune, one dried apricot, or ten raisins combined, and the authors were not able to discriminate between them [191]. These results open up a potential opportunity to further study the effects of raisins on telomere length.

The following section will provide a summary of evidence from human studies investigating grape effects in cardiometabolic and aging health, with an emphasis on interventions conducted in clinically relevant populations within the last 5 years, utilizing whole grape powders, preparations, wines, and extracts, given that reviews focusing on specific grape-derived compounds such as resveratrol have been reviewed elsewhere [193,194]. In line with past studies, recent trials have investigated the health benefits of a range of grape products, including grape pomace, juice and seed extracts, and grape seed oil and flour. Grape seed extract was the most common grape product used [47,195,196,197,198,199,200,201], followed by grape powder [48,202,203], with fewer studies utilizing real grapes, grape juice, and red wine [204,205,206,207,208,209]. Notably, one study utilized grape oil and another a grape seasoning, broadening its use and adding alternatives to include grapes in cooking [210,211].

Overall, recent studies varied to some degree in the grape product dose and study design. For studies utilizing grape seed extract, the dosage was mainly consistent, where the majority of studies used 300–400 mg/day [47,195,196,198,212,213], with the exception of two studies which utilized 520 mg/day [199] and 600 mg/day [200]. The average daily grape seed extract dose utilized in recent studies typically delivered about 286–572 mg gallic acid equivalent (GAE)—a frequently reported metric for estimations of total phenolic content of grapes and other antioxidant-rich dietary sources [196,200,213]. In contrast, concord grape juice can provide 592–636 mg GAE per serving [209]. For freeze-dried grape powder at 46 g/day, total polyphenol content varied considerably between studies, ranging from 21.9 to 163 mg GAE/day [201,203], which may reflect batch-to-batch variability in polyphenol composition. Grape pomace interventions ranged from approximately 96 mg GAE/day for a pomace seasoning [211] to 220–660 mg GAE/day for a grape pomace extract at long-term and acute doses, respectively [214]. In addition to variability in grape product dose and GAE values reported, recent intervention trials additionally varied in duration. The most commonly reported intervention timeframe was 4 weeks, with only two studies evaluating grape effects for 12 weeks [195,212]. Importantly, two studies investigated grape effects over longer duration, including a 4-month intervention conducted in healthy individuals with mildly elevated blood pressure [198], and a 6-month intervention conducted in a study population with chronic kidney disease (CKD) (including diabetic nephropathy and hypertension) [215].

Overall, the recent intervention trials reviewed in this section measured a variety of outcomes, such as blood sugar control, appetite, lipids metabolism biomarkers, oxidative stress, blood pressure, and clinical markers of inflammation—yet are lacking more in-depth, mechanistic, and comprehensive inflammatory and immune profiling. In general, the cardiometabolic results were positive, with some studies showing clear benefits [47,48,195,202,203], while others reported minimal [198,200,205,209,210,214,215] to no effects [201]. Given differences in baseline metabolic and inflammatory status between individuals characterized as healthy, having an elevated body weight status, and with cardiometabolic disease, we present an analysis of recent intervention trials within each of these clinically relevant populations. We additionally highlight studies conducted in populations within older age categories.

### 5.1. Results from Recent Intervention Trials Investigating Grape Effects on Biomarkers of Cardiometabolic Health and Aging in Healthy Populations

In line with mechanistic studies, grapes and grape-derived compounds have been shown to mediate antioxidant effects in healthy populations; however, the effects on inflammatory and immune markers—particularly those relevant to cardiometabolic disease risk—have been examined to a lesser degree, with limited evidence pointing to effects that are minimal or neutral [50]. However, outside the context of cardiometabolic disease and longevity, one recent study found that intake of red grape polyphenols for 3 months reduced serum concentrations of pro-inflammatory cytokines, while increasing anti-inflammatory IL-10 in subjects with allergic contact dermatitis to nickel [216]. Further, Notarnicola et al. [206] observed that while consumption of 5 g fresh table grape per kg of body weight/day did not impact serum CRP, glucose, aspartate aminotransferase (AST), alanine aminotransferase (ALT), or lipid panels, an increase in anti-inflammatory and insulin-sensitizing adiponectin was observed in healthy middle-aged adults. Interestingly, Segrestin et al. [197] observed that intake of a grape polyphenol-rich extract with 20% (m/m) of (pro)anthocyanidins (2 g/day polyphenols) in combination with an “overfeeding” diet (50% additional energy/high-fructose diet) reduced gynoid fat mass in female participants only. Thus, further studies into potential sex differences in response to grape intake are warranted.

Given the role of antioxidant activity in regulating inflammation, recent evidence suggests that the timing of grape intake may influence grape-related antioxidant effects. Blanton et al. [48] examined whether the time of day modified the impact of grape powder on oxidative stress markers following a high-fat meal. While urinary F2-isoprostane excretion, a marker of oxidative stress, increased in both morning and evening trials, greater postprandial antioxidant responses with grape powder were observed in the morning, suggesting potential chronobiological influences on polyphenol metabolism.

In addition to grapes mediating regulation of inflammation and immune profiles via antioxidant activity, grape-derived compounds may further modulate cardiometabolic disease and aging pathways by altering gut microbiota composition and metabolite formation, while an individual’s gut microbiota may further dictate grape-derived metabolite formation and health effects [217]. Dave et al. [202] investigated the effects of daily grape powder consumption on gut microbiota composition in healthy adults. Although no significant changes in overall α- or β- diversity were observed, grape consumption altered specific microbial taxa and urinary metabolites that may indicate beneficial shifts in microbial composition, suggesting that polyphenol-rich grapes may modulate gut microbiota composition and function. In contrast, Yang et al. [203], in a single-arm pilot study, reported improvements in gut microbial diversity and favorable changes in cholesterol and bile acid metabolism following grape powder supplementation, further supporting the potential for grape-derived polyphenols to promote cardiometabolic health via microbiome pathways. Similarly, Abrieux et al. [218] highlighted the potential role of wine-derived polyphenols in the maintenance of intestinal homeostasis. Consistently, Zorraquín-Peña et al. [204] reported that moderate red wine consumption altered gut microbial metabolites and reduced fecal water cytotoxicity, potentially decreasing colon cell damage risk and supporting intestinal health.

Similar to what is observed in mechanistic studies, grape’s benefits for CVD risk factors, such as serum lipids, further extends to blood pressure and vascular function in healthy populations. Schön et al. [198] evaluated grape seed extract supplementation in individuals with mildly elevated SBP and reported sex-specific responses, with significant reductions in both systolic and diastolic pressure observed in men, aligning with prior evidence that flavonoid-rich extracts can enhance endothelial function. Similarly, Nho and Kim [196] assessed grape seed extract in collegiate basketball players and found improvements in flow-mediated dilation, indicating enhanced endothelial responsiveness in an athletic population. Interestingly, a study comparing the effects of two vintages of the same red wine illustrated the complexity and variability inherent to evaluating a grape product such as wine. A three-arm crossover randomized controlled trial (RCT) examined vascular outcomes of 240 mL Japanese red wine (in two different vintages) or sparkling white grape juice as a control in 10 healthy adult men [219]. Only intake of the 2018 vintage reduced SBP and diastolic blood pressure (DBP) compared to the 2015 red wine. Authors explained that the 2018 vintage had doubled the amount of hydroxytyrosol than the 2015 vintage. Hydroxytyrosol is a phenolic compound considered among the most potent antioxidants after gallic acid [220]. Together, these findings highlight a consistent vascular benefit of grape seed extract across both general and athletic populations.

While intervention studies investigating the effects of whole grape preparations on inflammaging, senescence, and immune profiles are lacking, a number of recent studies report cognitive benefits. Rodrigo-Gonzalo et al. [221] reported that daily supplementation with 50 g of raisins for six months in healthy adults over 70 years of age improved certain domains of cognitive performance, quality of life, and functional activities, providing evidence of benefits in an aging population. Similarly, Amone et al. [222] demonstrated that 250 mg/day of a standardized grape extract administered over 12 weeks enhanced both short- and long-term cognitive performance in older adults in a randomized, double-blind, placebo-controlled trial. In contrast, Bell et al. evaluated whether 400 mg/day of grape seed extract was associated with cognitive benefits following a single acute dose and chronically following repeated daily dosage over 12 weeks (with mid-point testing at 6 weeks), in 60 young healthy adults. Researchers did not find consistent improvements in cognitive function in this population, suggesting that young healthy individuals may be less sensitive to grape seed extract than older or cognitive impaired populations. Importantly, in a pilot double-blinded parallel clinical dietary intervention, researchers showed that healthy postmenopausal women consuming 46 g of freeze-dried grape powder demonstrated a significant increase in hand grip strength and faster gait speed on a four-meter walk test. They also found a significant positive association between increase in grip strength and increase in plasma irisin levels within the grape group, indicating improved neuromuscular quality and potential mitigation of sarcopenia [223]. Together, these findings support a growing body of evidence that grape-derived products may exert cognitive effects across the lifespan, with the most consistent neuroprotective and biomechanical benefits observed in older populations.

An overview of recent intervention trials reporting the effects of grapes and grape-derived components on indicators of cardiometabolic health and aging in healthy populations (those defined by not meeting BMI classifications for overweight or obesity or exhibiting cardiometabolic disease diagnoses or risk factors based on reported descriptions of study populations) is presented below in Table 1.

### 5.2. Results from Recent Intervention Trials Investigating Grape’s Effects on Biomarkers of Cardiometabolic Health and Aging in Overweight and Obesity Populations

While mechanistic studies utilizing animal models of obesity have demonstrated robust antioxidant and anti-inflammatory effects, the findings from human intervention trials in populations with overweight and obesity have been more variable. Recent studies have evaluated both post-prandial and longer-term effects. Interestingly, a study using grape pomace extract after a high-fat meal showed that the effects depended on BMI: in normal-weight women, it reduced lipid peroxidation and protein oxidation, while in women with overweight or obesity, it reduced only protein oxidation [214]. However, another study testing grape powder in obese adults found no significant changes in post-meal blood sugar, lipids, or antioxidant capacity [201]. The grape powder provided in this trial was about 22 mg GAE per dose, a relatively low amount compared with the other studies. These differences help to explain why Garcia-Diez et al. [201] only observed modest postprandial effects, whereas interventions using higher doses often show more clear benefits. It is important to note that in a 12-week study, grape seed extract taken along with a calorie-restricted diet lowered anthropometric measurements and systemic inflammatory markers in adults with BMI between 25 and 40 kg/m^2^ [47], whereas 1 week of supplementation with 600 mg of grape seed extract per day in young men with a BMI over 25 resulted in decreased systolic blood pressure and aortic stiffness [200], suggesting that both acute and habitual intake of grape-derived products may confer cardiometabolic benefits.

A recent intervention trial conducted in men and women with overweight and obesity further examined the effects of grape products on blood glucose control and appetite regulation [209]. A randomized crossover trial tested Concord grape juice and found that its effects depended on both the polyphenol content and the flavor profile. Concord grape juice consumption increased urinary excretion of several phenolic metabolites compared with polyphenol-free beverages. Participants who excreted higher levels of a certain polyphenol metabolite had lower blood sugar levels after drinking the Concord juice compared to participants who excreted lower levels. When taken with a meal, the juice and a flavor-matched drink reduced hunger and the desire to eat compared with a reduced-flavor beverage [209]. This points to both the bioactive components and sensory properties of grapes as important factors in driving cardiometabolic benefits.

An overview of recent intervention trials reporting the effects of grapes and grape-derived components on indicators of cardiometabolic health in overweight and obesity populations, based on BMI reported in the primary research articles, is presented below in Table 2. It is important to note that recent studies investigating the cardiometabolic effects of grape products in populations with overweight and obesity are limited to study populations with a relatively younger average and range of age and that evaluation of aging-specific inflammatory, immune, or senescence markers is lacking. These factors are critical to investigate, given the well-established age-related changes in adiposity, and obesity-driven adipose dysfunction can promote inflammaging and increase chronic inflammation and cardiometabolic disease risk in younger adults [105,224]. While the preclinical evidence for the anti-inflammatory and immunomodulatory effects of grape products is strong, anti-inflammatory effects of freeze-dried whole grape powder have additionally been shown to reduce plasma TNF-α in both pre- and post-menopausal women with an average BMI in the obesity range, suggesting that more in-depth analysis of inflammatory pathways in current studies is warranted [185].

### 5.3. Results from Recent Intervention Trials Investigating Grape Effects on Biomarkers of Cardiometabolic Health and Aging in Populations with Cardiometabolic Disease or Increased Cardiometabolic Disease Risk

A larger proportion of recent studies evaluating the effects of grape products on cardiometabolic disease markers have focused on populations with diagnosed cardiometabolic disease or conditions characterized by increased cardiometabolic disease risk, based on well-established metrics and study population descriptives reported by the primary research articles (e.g., hyperlipidemia, T2DM, elevated blood pressure, etc.) (Table 3). Notably, a number of studies included populations or subgroups with a higher average age relative to the research conducted in healthy and overweight/obesity populations. However, consistent with these recent studies, limited analyses of inflammatory, immune function or aging biomarkers have been carried out, and the effects of grape products on cardiometabolic risk factors are variable.

Consistent with earlier intervention studies, positive effects of grape products on blood pressure were observed in three out of five of the recent reviewed studies. A reduction in DBP was seen with 5 weeks of raisins (~916 mg GAE/day) [208] and the 1 week and 300 mg of grape seed extract intervention (~286 mg GAE/day) used by Lira et al. [213], while 6 weeks of grape pomace seasoning (~96 mg GAE/day) [211] resulted in diastolic and systolic blood pressure reduction. Blood pressure changes observed in these studies corresponded to improvements in other cardiometabolic and oxidative stress measures, such as increases in TAC and reductions in MDA and fasting glucose [208,211]. The length of the intervention may explain the different blood pressure outcome, when more time may be needed for effects to show (independent of the dosage). This is also supported by another study that lasted 12 weeks, where the combined bilberry and grape seed extract (300 mg/d) reduced SBP and DBP [212]. The positive effects of grapes on blood pressure shown in the present review are consistent with systolic blood pressure results from a previous meta-analysis by Ashoori et al. [226], evaluating the effects whole grapes and their products on blood pressure and endothelial function. They reported that consumption of grape products reduced SBP. However, no beneficial effects were observed on DBP, endothelial function, heart rate, and pulse rate [226].

Inconsistent results have additionally been reported with regard to grape product effects on serum lipids. For example, a 4-week intervention study found that grape seed oil reduced LDL-C in hypercholesterolemic adults but did not significantly affect LDL-C or HDL-C in healthy adults [210]. Further, in adults with non-alcoholic fatty liver disease (NAFLD), daily intake of grape seed extract (520 mg/day) for 8 weeks promoted favorable decreases in total cholesterol, triglycerides, LDL-C, LDL/HDL ratio, and the atherogenic index of plasma (AIP), while increasing HDL-C [199]. However, there were no effects of grapes on serum lipids or measures of lipid metabolism in other recent studies conducted in a population with cardiometabolic disease or increased cardiometabolic disease risk that were identified in this review (most of which used a diverse range of grape products over different time periods and varied in baseline health status) [208,211,212,225]. Together, these findings suggest that grape seed-derived products may be helpful in lowering plasma LDL-C lipids in certain populations, but the degree of benefit may depend on the form and duration of the product used and the cardiometabolic profile of the individual.

Baseline health and grape preparation may further explain differences observed in grape effects on inflammatory markers. Recent studies report minimal to no effect of grape products on inflammatory markers such as hsCRP, with changes failing to reach statistical significance. However, in line with mechanistic cell- and animal-based studies, grape products have shown to be anti-inflammatory in earlier intervention research. In a randomized, placebo-controlled, triple-blind, one-year trial, 35 older male patients (with a mean age of ~60 years) with type 2 diabetes, hypertension, and stable coronary artery disease received either placebo (maltodextrin), conventional grape extract without resveratrol, or grape extract enriched with resveratrol. Both grape extracts’ total phenolic content was very similar and administered once daily for 6 months, then twice daily for 6 months, while participants maintained their diet and medications and avoided other grape-derived products. Compared to placebo, serum alkaline phosphatase was significantly reduced in both grape extract and grape extract + resveratrol groups. Further, grape extract + resveratrol supplementation reduced serum IL-6 levels, whereas grape extract without resveratrol showed smaller, non-significant changes, providing preliminary evidence of reduction in pro-inflammatory markers in this older, high-risk population [49]. Similarly, in men classified with dyslipidemia and metabolic syndrome, daily intake of a whole grape powder for 4 weeks increased plasma concentrations of anti-inflammatory adiponectin and IL-10, whereas no effects were observed in metabolic syndrome participants without dyslipidemia [186].

Importantly, grape-derived products have also shown benefits in intervention trials conducted with participants with diabetic kidney disease, which is known to significantly increase CVD risk. In one six-month supplementation and weight loss study, high-dose grape seed flour was safe and well tolerated by participants with chronic kidney disease and led to improvements in kidney function measures, proteinuria, and blood pressure. The intervention also appeared to slow disease progression, with a greater proportion of participants demonstrating stable or improved chronic kidney disease status compared with the placebo group. While inflammation and anemia markers did not improve significantly, the authors noted that the intervention was associated with improvements in corrected weight loss, although baseline body weight was not reported. Overall, these results suggest a potential role for grape seed flour in slowing disease progression, especially in diabetic kidney disease [215]. In contrast, earlier research has reported anti-inflammatory effects of grapes in CKD. Janiques et al. [227] conducted a randomized, double-blind, placebo-controlled trial in non-diabetic hemodialysis patients (n = 32, mean age ~53 years) to assess the effects of grape powder supplementation (500 mg polyphenols/day) on inflammatory and antioxidant markers. After five weeks, participants receiving grape powder demonstrated a significant increase in GPX activity and maintained stable hsCRP plasma levels, whereas hsCRP increased in the placebo group. These findings suggest that, depending on the grape product and complexity of CKD cases, grape-derived polyphenols may exert antioxidant and anti-inflammatory effects in populations with chronic kidney disease, highlighting their potential to modulate pathways relevant to cardiometabolic health and systemic inflammation.

Given the role of the gut microbiome in mediating grape effects based on mechanistic studies, the gut microbiota was assessed in some of the recent studies reviewed, which reported modest changes in the type/abundance of the microbiome [207,212,225]. In a study by Haas et al. [207], there were no differences in TMAO with 3 weeks of red wine in men with coronary artery disease. However, there were significant changes in some gut microbiota species, including *Ruminococcaceae, Prevotella, Roseburia* and several *Bacteroides* species [207]. In a 6-week crossover intervention in 49 adults with obesity and at least two characteristics of metabolic syndrome, Ramos-Romero et al. [225] found that subjects classified as responders (defined as those whose fasting insulin responses improved after grape pomace intake) had lower baseline levels of *Firmicutes*, *Prevotella*, and the fecal SCFA butyrate, yet higher serum expression of miR-222, suggesting that the beneficial effects of grape pomace on insulin sensitivity may be greater in individuals with less favorable gut microbiota profiles at baseline. The results from the studies presented in Table 3 support the role of grapes in changing the gut microbiome homeostasis. This is consistent with a thorough review describing how microbiota-targeted interventions can optimize metabolic health [217].

### 5.4. Summary: Results from Recent Intervention Trials Investigating Grape Effects on Biomarkers of Cardiometabolic Health and Aging Across Populations

Overall, recent studies complement the existing literature to demonstrate both neutral and positive effects of grape products on cardiometabolic disease markers, with recent studies expanding scientific knowledge of grape effects on more diverse grape preparations, study populations, and gut microbiome parameters. Although the interventions, populations, and measured outcomes varied, these studies collectively demonstrate the potential for grape-derived products to support vascular health by reducing blood pressure, improve lipid metabolism in select populations, modulate the gut microbiome and associated metabolome, and mitigate oxidative stress.

While the current evidence elucidating the effects of grapes in human studies provides valuable insights, there are several limitations to consider. First, many of the trials had small sample sizes (n = 10–25) [196,200,201,204,210,212,213,214,219]. The length of the interventions varied widely, from a single dose to six months, and the grape-based products used differed in form, composition, and polyphenol content (e.g., ranging from ~22 to ~916 mg GAE/day) [201,208]. This variation makes it difficult to compare results across studies or to identify the specific components responsible for the observed effects. In some studies, lifestyle and dietary factors outside of the intervention period were not well controlled [201,209,214], and very few studies included long-term follow-up or clinical endpoints [47,200,215]. Further, the scope of outcome measures is relatively limited to traditional measures of cardiometabolic disease risk; thus, incorporating ex vivo or omics-based assessments of grape effects may provide better mechanistic insight into the effects of grapes and grape-derived compounds on human health and help explain interindividual variability across or within studies.

Accordingly, the majority of recent studies also lacked details on demographics and social determinants of health, which would help to better characterize the population studied. Including these details could better inform whether it is possible to generalize the studies’ conclusions. However, it is important to note that the recent studies investigating grape effects in cardiometabolic disease/higher risk populations had an overrepresentation of male participants and a higher mean age compared to studies investigating grape effects in populations classified as healthy and with overweight or obesity. Finally, differences in how individuals absorb and metabolize polyphenols may have influenced the results, which could explain some of the observed variability in responses between participants [209].

## 6. Conclusions

Taken together, compelling evidence from mechanistic cell- and animal-based studies demonstrates that grape-derived products and bioactives may alleviate physiological complications associated with various cardiometabolic diseases and aging via modulation of cellular inflammatory, metabolic, and antioxidant pathways and the gut microbiome, thereby reducing pro-inflammatory mediators and immune-mediated responses, metabolic tissue dysfunction, and inflammaging and senescence profiles. While some evidence from human intervention trials support these findings, the use of diverse grape products with unique compositions and/or alcohol content (e.g., wine), different study designs and participant populations, and limited translational mechanistic measures make it difficult to assess the full extent of grape effects on the inflammatory and immune-mediated mechanisms underlying cardiometabolic disease risk and healthy aging. Thus, additional studies in human subjects are warranted to identify determinants of grape product-specific and the individual-specific benefits of grapes, given promising effects observed across study models.

## Figures and Tables

**Table 1:** Overview of recent findings from intervention trials investigating grape effects cardiometabolic health and aging in healthy populations.

Study	Design	Sample (N and Male/Female)	Population	Intervention	Duration	Results
Bell et al. (2022) [195] U.K.	Acute-on-chronic randomized, double-blind, placebo-controlled	60 (9 M/51 F)	Healthy adults (age 18–30 y)	Grape seed polyphenol extract (400 mg/day) (mg GAE/day not reported)	Acute: 6 h Chronic: 12 w	↔ Cognitive function; Acute: ↓ Switching Task RT (faster) for treatment vs. placebo (2–4 h); placebo better on AVLT Chronic: Simple finger tapping maintained vs. placebo decline; ↑ recall (6→12 w); ↑ RT (slower) in some tasks; ↑ mood (both groups)
Blanton et al. (2023) [48] U.S.	2 × 2 factorial, randomized, double-blind, placebo-controlled	32 (13 M/19 F)	Healthy adults (age 18–50 y)	Freeze-dried grape powder (46 g) + high-fat meal challenge; 2-day low-antioxidant diet (mg GAE/day not reported)	One dose/single dose	↓ Postprandial urinary F2-isoprostanes (grape vs. placebo; morning only)
Charoenwoodhipong et al. (2023) [219] U.S.	Randomized, 3-arm, single-blind, controlled crossover	10 (10 M/0 F)	Healthy adult men (age 50–70 y)	240 mL Zweigelt red wine (2015 or 2018 vintage); 24-h low polyphenol diet 2015 wine: 674 mg GAE/serving 2018 wine: 583 mg GAE/serving	One dose/single dose (7-day washout between arms)	↓ Arterial stiffness with both wines vs. control ↓ SBP and DBP with 2018 wine only vs. 2015 wine and control
Dave et al. (2023) [202] U.S.	Single-arm, crossover	29 (gender breakdown nor reported)	Healthy adults (age 19–55 y)	Grape powder (36 g/day equivalent to 3 servings of grapes/day; 1 serving = 1.5 cups fresh grapes) + restricted diet (low polyphenol diet) (mg GAE/day not reported)	2 w intervention; 2 w run-in and 4 w washout	Vs. baseline: ↔ α- and β-diversity; trend ↑ *Streptococcus thermophilus*; ↓ *Holdemania* spp.; ↑ urinary metabolites (eg., tartaric acid) following grape consumption
Nho and Kim (2022) [196] South Korea	Randomized, double-blind, placebo-controlled crossover	12 (sex not reported)	Healthy division I collegiate basketball players (mean age 20.2 ± 1 y)	Grape seed extract (1 × 300 mg/day) (~286 mg GAE/day)	2 w per treatment with 1 w washout	Vs. placebo: ↑ Flow mediated dilation
Schön et al. (2021) [198] Germany	Randomized, double-blind, placebo-controlled	80 (45 M/35 F)	Mildly elevated BP (age 40–70 y; women: postmenopausal)	Grape seed extract (2 × 150 mg/day) (mg GAE/day not reported)	16 w	Vs. placebo: ↔ Overall BP; ↓ SBP and DBP in men only; ↑ nocturnal dipping in women
Segrestin et al. (2022) [197] France Switzerland	Randomized, double-blind, placebo-controlled parallel-group	61 (42 M/19 F)	Healthy adults (mean age: men: 31 ± 2 y and women: 26 ± 1 y)	Grape polyphenol extract (2000 mg/day polyphenols) + high calorie-high fructose overfeeding diet with 50% energy excess mg GAE/day not reported)	31 d	Vs. placebo: ↔ insulin sensitivity (GIR, HOMA-IR), or metabolic adaptations ↓ gynoid fat mass accumulation and gluteofemoral adipocyte size in women
Notarnicola et al. (2022) [206] Italy	Randomized, controlled parallel- group	40 (13 M/27 F)	Healthy adults (Grape group: Mean age 47.4 ± 9.5 y; Control group: 44.1 ± 10.1 y)	Fresh table grapes (5 g/kg BW) vs. no grape consumption + restriction of alcohol, caffeine and polyphenol-rich foods (mg GAE/day not reported)	3 w intervention with 4 w follow-up	Vs. baseline: ↑ Omega-3 index (EPA+DHA) and adiponectin (maintained at follow-up); ↓ FGF21; ↔ CRP, glucose, AST, ALT and lipid panel
Yang J. et al. (2021) [203] U.S.	Two-phase dietary intervention	19 (6 M/13 F)	Healthy adults (age 21–55 y)	Freeze-dried grape powder (46 g) + low polyphenol, low fiber diet (~163 mg GAE/day)	4 w standardization with 4 w intervention	Vs. baseline: ↑ alpha diversity, ↔ beta-diversity; ↑ abundance of Verrrucomicrobia; ↑ *Akkermansia, Flavonifractor*, and *Lachnospiraceae*; ↓ *Bifidobacterium* and *Dialister*; ↓ TC, HDL and total bile acids; ↓ trend LDL; ↔ TG

Table abbreviations: ALT: alanine aminotransferase; AST: aspartate aminotransferase; AVLT: auditory verbal learning test; BP: blood pressure; BW: body weight; CRP: C-reactive protein; DBP: diastolic blood pressure; DHA: docosahexaenoic acid; EPA: eicosapentaenoic acid; F: female; FGF21: fibroblast growth factor 21; GAE: gallic acid equivalents; GIR: glucose infusion rate; HDL: high-density lipoprotein cholesterol; HOMA-IR: homeostatic model assessment of insulin resistance; LDL: low-density lipoprotein cholesterol; M: male; RCT: randomized controlled trial; RT: response time; SBP: systolic blood pressure; TC: total cholesterol, TG: triglycerides. ↑: increase (grape group vs. control, unless other noted); ↓: decrease (grape group vs. control, unless other noted); ↔: no change/difference (grape group vs. control, unless other noted). GAE values reported are only those reported by the primary reference cited.

**Table 2:** Overview of grape effects in populations with overweight and obesity from recent clinical trials.

Study	Design	Sample (N and Male/Female)	Population	Intervention	Duration	Results
Choleva et al. (2022) [214] Greece	Randomized, double-blind, placebo-controlled crossover	18 (0 M/ 18 F)	Apparently healthy women (age 22–35 y; BMI < 25 and >25 kg/m^2^)	Group 1 (postprandial): Grape pomace extract (6 capsules = 3.5 g) + high-fat meal (660 mg GAE/day) Group 2 (pilot long-term): Grape pomace extract (2 capsules = 1.2 g) (220 mg GAE/day)	Group 1: Single dose Group 2: 28 d	Overall: ↓ postprandial TG at 300 min; ↔ glucose, insulin, GPx BMI < 25 kg/m^2^: ↓ protein carbonyls, TBARS, SOD activity ↓ UA response BMI > 25 kg/m^2^: ↓ protein carbonyls ↑ UA response vs. placebo at selected postprandial timepoints
Coelho et al. (2021) [209] U.S.	Randomized, double-blind, three treatment arms crossover trial	68 (42 M/26 F)	Adults (age: 25–60 y; BMI: 25–34.9 kg/m^2^)	CGJ, LPF, and LP either without (trial I) or with (trial II) a meal + low phenolic diet Trial I: 592 mg GAE/day Trial II: 636 mg GAE/day	3 × 4 d crossover periods with 6 d washouts	Trial I: ↔ postprandial glycemia, appetite and cognitive function among treatments Trial II: ↓ hunger, desire to eat and prospective consumption with CGJ and LP vs. LPF; ↔ postprandial glycemia, other appetite measures and cognitive function among treatments
Dillon et al. (2022) [200] U.S.	Randomized, double-blind, placebo-controlled crossover	10 (10 M/ 0 F)	Adults (age 18–27 y; BMI > 25 kg/m^2^; WC >90 cm)	Grape seed extract (2 capsules = 600 mg) (~572 mg GAE/day)	1 w	Vs. placebo: ↓ MAP, DBP at rest and across workloads; ↓ SBP at rest and 40% VO2 max; ↓ resting aortic stiffness; ↔ HR, SV, Q and TVC
García-Díez et al. (2021) [201] Spain	Randomized, double-blind, placebo-controlled crossover	25 (10 M/15 F)	Adults with obesity (age 18–65 y; BMI ≥30–<40 kg/m^2^)	Freeze-dried grape powder (46 g; =252 g fresh grapes) + high-fat, high-sugar breakfast; high-fat, high-sugar lunch provided 5 h later (~22 mg GAE/day)	Single dose	Vs. placebo: Metabolic: ↔ postprandial glucose, insulin, TG, UA vs. placebo Oxidative stress: ↔ plasma antioxidant capacity Satiety: ↔ appetite
Parandoosh et al. (2020) [47] Iran	Randomized, double-blind, placebo-controlled	40 (7 M/33 F)	Adults (Age 20–50 y with a BMI of 25–40 kg/m^2^)	Grape seed extract (300 mg/day) + calorie-restricted diet (mg GAE/day not reported)	12 w	Vs. placebo: ↓ Anthropometric measurements (body weight, BMI, WC, HC); ↔ WHR, body fat and lean body mass; ↓ hs-CRP, TNF-α; ↓ NPY; ↔ appetite

Table Abbreviations: BMI: body mass index; CGJ: Concord Grape Juice; DBP: diastolic blood pressure; GAE: gallic acid equivalents GPx: glutathione peroxidase; HC: hip circumference; HR: heart rate; hs-CRP: high-sensitivity C-reactive protein; LP: phenolic-free grape flavored drink; LPF: low-flavor intensity (essence) grape flavored drink; MAP: mean arterial pressure; NPY: neuropeptide Y; Q: cardiac output; SBP: systolic blood pressure; SOD: superoxide dismutase; SV: stroke volume; TBARS: thiobarbituric acid reactive substances; TG: triglycerides; TNF-α: tumor necrosis factor-alpha; TVC: total vascular conductance; UA: uric acid; VO_2_: oxygen consumption; WC: waist circumference; WHR: waist-to-hip ratio. ↑: increase (grape group vs. control, unless other noted); ↓: decrease (grape group vs. control, unless other noted); ↔: no change/difference (grape group vs. control, unless other noted). GAE values reported are only those reported by the primary reference cited.

**Table 3:** Overview of grape effects in populations with elevated cardiometabolic disease risk from recent clinical trials.

Study	Design	Sample (N(M/F))	Population	Intervention	Duration	Results
Pazos-Tomas et al. (2021) [210] Mexico	Randomized, longitudinal, prospective quasi-experimental pretest–posttest	Phase 1: 18 (12 M/6 F); Phase 2: 10 (3 M/7 F)	Phase 1: Healthy adults (18–30 y, BMI 18.5–24.9 kg/m^2^); Phase 2: Adults (18–45 y, BMI 25–29.9 kg/m^2^ with familial hypercholesterolemia)	10 mL/day avocado oil or grape seed oil (mg GAE/day not reported)	4 w	Within Grape seed oil vs. baseline: Phase 1: Healthy adults: ↓ Weight, BMI and WC; ↔ fasting glucose, HDL-C or LDL-C Phase 2: Adults with hypercholesterolemia: ↓ weight, BMI, WC and HC, fasting glucose and LDL-C ↔ HDL-C
Haas et al. (2022) [207] Brazil	Randomized, controlled crossover	42 (42 M/0 F)	Adults with CAD (age 46–69 y; BMI < 30 kg/m^2^)	Red wine (250 mL/day, 5 d/week with 24 g of alcohol and 2155 mg/L catechin equivalents) + restriction of polyphenol-rich and fermented foods (mg GAE/day not reported)	2 × 3 w crossover with 2 w washout	Vs. alcohol abstention control period: ↔ TMAO; ↑ predominance of Bacteroides, Ruminococcaceae, Roseburia, and Prevotella; altered plasma metabolome: amino acid, lipid, carbohydrate, vitamins/cofactor pathways
Grohmann et al. (2023) [212] Scotland	Randomized, double-blind, placebo-controlled crossover	14 (4 M/10 F)	Adults at risk of T2DM (age ≥ 45 y; BMI > 28 kg/m^2^, HbA1c ≥ 5.5% or diabetes risk score of 20–24 points)	Bilberry (250 mg) and grape seed extract (300 mg /day) (mg GAE/day not reported)	12 w in random order without a washout period	Vs. control: ↓ 24 h SBP, DBP; ↔ HbA1c, glucose, insulin, HOMA-IR, TC, LDL-C, HDL-C; ↑ Fusicatenibacter- related bacteria in BP responders
Lira et al. (2025) [213] U.S.	Randomized, double-blind, placebo-controlled crossover	10 (10 M/0 F)	Adults with elevated and stage 1 HT (18–30 y)	Grape seed extract (300 mg) (~286 mg GAE/day)	2 × 1 w crossover with 1 w washout	Vs. placebo: ↓ DBP and MAP; ↔ SBP, HR, SV, CO, HRV and cold pressor test responses
Ramos-Romero et al. (2021) [225] Spain	Randomized, controlled crossover	49 (27 M/22 F)	Adults with at least 2 factors of metabolic syndrome, classified as responders (n = 23) or non-responders (n = 26) according to fasting insulin response to grape pomace supplementation (mean age 43 ± 2 y)	Freeze-dried grape pomace (8 g/day) (mg GAE/day not reported)	2 × 6 w crossover with 2 w washout	Responders: ≥10% reduction in fasting insulin; ↓ Prevotella, Firmicutes; ↑ miR-222 serum expression vs. non-responders
Shishehbor, F et al. (2022) [208] Iran	Randomized controlled parallel	38 (13 M/25 F)	Adults with hyperlipidemia (TC > 200 mg/dL or TG > 200 mg/dL) (mean age 41 ± 10.4 y)	Black seed raisins (90 g/day) Control: routine dietary recommendation/no intervention (~916 mg GAE/day)	5 w	Vs. control: ↑ TAC; ↓ MDA, DBP; ↔ SBP, TC, LDL-C, HDL-C, TG, FBS, hsCRP
Taladrid et al. (2022) [211] Spain	Randomized, controlled, parallel	29 (12 M/17 F)	Adults (HRC: BP > 130/85 mmHg and/or fasting glucose > 100 mg/dL) and healthy adults	Grape pomace seasoning (2 g/day) + reduced sodium salt intake (~96 mg GAE/day)	6 w	Vs. baseline: Combined analysis (healthy + HRC): ↓ SBP, DBP, MBP, and fasting glucose; ↔ α-/β- diversity; ↑ protocatechuic acid; ↓ propionic acid ↔ acetic acid and butyric acid
Ghanbari, et al. (2024) [199] Iran	Randomized, double-blind, placebo-controlled	50 (24 M/26 F)	Adults with NAFLD (age: 20–60 y; BMI: 25–35 kg/m^2^; moderate-to-severe hepatic steatosis)	Grape seed extract (two 260 mg capsules) (mg GAE/day not reported)	8 w	Vs. control: ↓ Hepatic steatosis severity, ALT, AST, SBP, DBP, MAP, insulin, HOMA-IR, TC, TG, LDL-C, LDL/HDL ratio and AIP; ↑ HDL-C and QUICKi; ↔ FBS and HbA1c
Bejaoui et al. (2022) [215] Tunisia	Randomized, placebo-controlled	40 (22 M/18 F)	Adults with CKD stages 2–4 (including diabetic nephropathy and hypertension) (mean age: GSF: 57.9 ± 2.1 y, placebo: 58.4 ± 1.72 y)	Grape Seed Flour (~1 g/kg/day) (mg GAE/day not reported)	6 m	Within-group (GSF) vs. baseline: improved weight loss; ↑ CAT, GPx, SOD; ↓ MDA, protein carbonyls, H_2_O_2_, free iron, BP; ↔ HbA1c, glycemia, hematology and lipidemia Vs. placebo: ↑ GFR; ↓ urine protein/creatinine ratio; improved/stabilized CKD stage progression

Table abbreviations: AIP: atherogenic index of plasma; ALT: alanine aminotransferase; AST: aspartate aminotransferase; BMI: body mass index; BP: blood pressure; CAD: coronary artery disease; CAT: catalase; CKD: chronic kidney disease; CO: cardiac output; DBP: diastolic blood pressure; FBS: fasting blood glucose; GAE: gallic acid equivalents; GFR: glomerular filtration rate; GPx: glutathione peroxidase; GSF: grape seed flour; HbA1c: glycated hemoglobin; HC: hip circumference; HDL-C: high-density lipoprotein cholesterol; HOMA-IR: homeostatic model assessment of insulin resistance; HRC: high cardiometabolic risk; HR: heart rate; HRV: heart rate variability; hsCRP: high-sensitivity C-reactive protein; HT: hypertension; H_2_O_2_: hydrogen peroxide; LDL-C: low-density lipoprotein cholesterol; MAP: mean arterial pressure; MBP: mean blood pressure; MDA: malondialdehyde; NAFLD: non-alcoholic fatty liver disease; QUICKi: quantitative insulin sensitivity check index; SBP: systolic blood pressure; SOD: superoxide dismutase; SV: stroke volume; TAC: total antioxidant capacity; TC: total cholesterol; TG: triglycerides; TMAO: trimethylamine *N*-oxide; T2DM: type 2 diabetes mellitus; WC: waist circumference. ↑: increase (grape group vs. control, unless other noted); ↓: decrease (grape group vs. control, unless other noted); ↔: no change/difference (grape group vs. control, unless other noted). GAE values reported are only those reported by the primary reference cited.

## Data Availability

No new data were created or analyzed in this study. Data sharing is not applicable to this article.

## Abbreviations

The following abbreviations are used in this manuscript:

4-HDA	4-hydroxyalkenals
β-gal	β-Galactosidase
AhR	Aryl hydrocarbon receptor
AIP	Atherogenic index of plasma
AKT	Protein kinase B
ALT	Alanine aminotransferase
AMPK	AMP-activated protein kinase
AP-1	Activator protein-1
Apo A-I	Apolipoprotein AI
AST	Aspartate aminotransferase
AVLT	Auditory Verbal Learning Test
BAX	Bcl-2-associated X protein
BMI	Body mass index
BP	Blood pressure
BW	Body weight
CAD	Coronary artery disease
CAT	Catalase
CCR2	C-C motif chemokine receptor 2
CD	Cluster of differentiation
CGJ	Concord grape juice
CKD	Chronic kidney disease
CO	Cardiac output
COX-2	Cyclooxygenase-2
CRP	C-reactive protein
CVD	Cardiovascular disease
DAMPs	Damage-associated molecular patterns
DBP	Diastolic blood pressure
DHA	Docosahexaenoic acid
DOCA	Deoxycorticosterone acetate
EPA	Eicosapentaenoic acid
eNOS	Endothelial nitric oxide synthase
ERK	Extracellular signal-regulated kinase
eWAT	Epididymal white adipose tissue
FBS	Fasting blood glucose
FFAs	Free fatty acids
FGF21	Fibroblast growth factor 21
FMD	Flow-mediated dilation
GAE	Gallic acid equivalents
GFR	Glomerular filtration rate
GIR	Glucose infusion rate
GLUT4	Glucose transporter type 4
GPX	Glutathione peroxidase
GSEA	Gene Set Enrichment Analysis
GSF	Grape seed flour
GSIS	Glucose-stimulated insulin secretion
HbA1c	Glycated hemoglobin
HC	Hip circumference
HDL	High-density lipoprotein
HDL-C	High-density lipoprotein cholesterol
HFD	High-fat diet
HFFD	High-fat-fructose diet
HFF	High-fat-fructose-fed
HO-1	Heme oxygenase-1
HOMA-IR	homeostatic model assessment of insulin resistance
HR	Heart rate
HRC	High cardiovascular risk
HRV	Heart rate variability
HSCs	Hematopoietic stem cells
hsCRP	High-sensitivity C-reactive protein
HT	Hypertension
HUVECs	Human umbilical vein endothelial cells
H_2_O_2_	Hydrogen peroxide
ICAM-1	Intercellular adhesion molecule-1
IFN-γ	Interferon-gamma
IKK	Inhibitor of κB kinase
IκBα	Inhibitor of κB alpha
IL-1β	Interleukin-1 beta
IL-1R	Interleukin-1 receptor
IL-6	Interleukin-6
IL-10	Interleukin-10
IL17R	Interleukin 17 receptor
iNOS	Inducible nitric oxide synthase
IRS1	Insulin receptor substrate-1
ISO	Isoproterenol
JAK	Janus kinase
JNK	c-Jun *N*-terminal kinase
LDL	Low-density lipoprotein
LDL-C	Low-density lipoprotein cholesterol
LP	Phenolic-free grape flavored drink
LPF	Low-flavor intensity (essence) grape-flavored drink
LPS	Lipopolysaccharide
MAP	Mean arterial pressure
MAPK	Mitogen-activated protein kinase
MBP	Mean blood pressure
MASLD	Metabolic dysfunction-associated steatotic liver disease
MCP-1	Monocyte chemoattractant protein-1
MDA	Malondialdehyde
MI	Myocardial infarction
MMP3	Matrix metalloproteinase-3
MPO	Myeloperoxidase
MSCs	Mesenchymal stem cells
MT	Million tonnes
NAD	Nicotinamide adenine dinucleotide
NADPH	Nicotinamide adenine dinucleotide phosphate
NAFLD	Non-alcoholic fatty liver disease
NAMPT	Nicotinamide Phosphoribosyltransferase
NF-κB	Nuclear factor kappa B
NLRP3	NOD-like receptor family pyrin domain-containing 3
NO	Nitric oxide
NOS	Nitric oxide synthase
NPY	Neuropeptide Y
Nrf2	Nuclear factor erythroid 2-related factor 2
OxLDL	Oxidized low-density lipoprotein
ORAC	Oxygen radical absorbance capacity
PBMC	Peripheral blood mononuclear cells
PGC-1α	Peroxisome proliferator-activated receptor gamma coactivator 1-alpha
PI3K	Phosphoinositide 3-kinase
PPARs	Peroxisome proliferator-activated receptors
PTP-1B	Protein tyrosine phosphatase 1B
Q	Cardiac output
QUICKi	Quantitative insulin sensitivity check index
RCT	Randomized controlled trial
RNS	Reactive nitrogen species
ROS	Reactive oxygen species
RPE	Retinal pigment epithelial cells
RT	Response time
SAA	Serum amyloid A
SASP	Senescence-associated secretory phenotype
SBP	Systolic blood pressure
SCFAs	Short-chain fatty acids
SHR	Spontaneously hypertensive rats
SIRT1	Sirtuin 1
SOCS3	Suppressor of cytokine signaling 3
SOD	Superoxide dismutase
STAT1	Signal transducer and activator of transcription 1
STAT3	Signal transducer and activator of transcription 3
SV	Stroke volume
T2DM	Type 2 diabetes mellitus
TAC	Total antioxidant capacity
TBARS	Thiobarbituric acid reactive substances
TEER	Transepithelial/transendothelial electrical resistance
Th	T helper cell
TG	Triglycerides
TGF-β	Transforming growth factor-beta
TGR5	Takeda G-protein-coupled receptor 5
TIMP-2	Tissue inhibitor of metalloproteinases 2
TLR	Toll-like receptors
TMA	Trimethylamine
TMAO	Trimethylamine *N*-oxide
TNF-α	Tumor necrosis factor-alpha
TNFR1	Tumor necrosis factor-alpha receptor 1
TPC	Total polyphenol content
Treg	Regulatory T cell
TVC	Total vasculature conductance
UA	Uric acid
USDA	United States Department of Agriculture
VAT	Visceral adipose tissue
VCAM-1	Vascular cell adhesion molecule-1
VO_2_	Oxygen consumption
VSMCs	Vascular smooth muscle cells
WC	Waist circumference
WPF	Wine pomace fraction after colonic fermentation
WPG1	Wine pomace fraction after gastrointestinal digestion
ZO-1	Zonula occludens-1
